# Research Partnerships in Rural and Remote Virtual Health Innovation Projects: A Scoping Review

**DOI:** 10.1111/ajr.70220

**Published:** 2026-06-08

**Authors:** Vania Tjandra, Jason Yi, Emily Thomson, Jenna Benbaruj, Carolyn Canfield, Anurag Singh, Jane Jun, Femke Hoekstra

**Affiliations:** ^1^ Centre for Chronic Disease Prevention and Management, The University of British Columbia Kelowna British Columbia Canada; ^2^ Faculty of Arts and Social Sciences, Department of Psychology The University of British Columbia Kelowna British Columbia Canada; ^3^ Faculty of Medicine, The University of British Columbia Vancouver British Columbia Canada; ^4^ Department of Family Practice The University of British Columbia Vancouver British Columbia Canada; ^5^ Division of Medical Science The University of Northern British Columbia Prince George British Columbia Canada; ^6^ Department of Medicine, Division of Nephrology The University of British Columbia Vancouver British Columbia Canada; ^7^ Southern Medical Program, Library Services, The University of British Columbia Kelowna British Columbia Canada; ^8^ Department of Medicine, Division of Social Medicine University of British Columbia Vancouver British Columbia Canada

**Keywords:** collaborative research, community‐engaged research, digital health app, health equity, knowledge co‐production, research partnership, rural and remote health, telehealth, virtual health innovation

## Abstract

**Introduction:**

Partnerships with research users (e.g., communities, clinicians) are effective in supporting virtual health innovation (VHI) projects to co‐develop, implement and evaluate sustainable, relevant innovations that improve care access in rural communities. However, building trusting relationships with underrepresented groups is challenging and time‐intensive.

**Objective:**

To synthesise partnership processes (principles, strategies) and effects (outcomes/impacts) from rural VHI projects.

**Design:**

A scoping review of peer‐reviewed articles on rural VHI partnership projects (2020–2024) identified through five databases. Partnership principles (norms/beliefs), strategies (observable actions) and outcomes/impacts (short‐ to long‐term effects) were extracted and analysed using directed content analysis. Reporting followed the PRISMA‐ScR checklist.

**Findings:**

Of 7413 abstracts, 85 articles met inclusion criteria. VHI partnership projects were conducted in 15 countries, covering telehealth, digital education and health apps. Nearly half (45.9%) involved partnerships with Indigenous communities. We identified 149 principles and 80 strategies. Principles commonly related to relationship building (55.3%), shared decision‐making (51.8%) and knowledge co‐production (49.4%). From nine evaluation studies, 36 outcomes/impacts were extracted, of which 81% were positive.

**Discussion:**

While a range of partnership processes and effects were identified, reporting was inconsistent, and few studies incorporated formal evaluations. Future research should adopt systematic approaches to report and evaluate their partnership engagement, thereby expanding the evidence base and facilitating shared learning for more effective and meaningful engagement with rural communities.

**Conclusion:**

This systematic overview of partnership processes and effects lays the groundwork for more effective, culturally safe engagement. Strengthening partnerships in these contexts can improve healthcare access and reduce rural health disparities.

## Abstract (highlights)

**What Is Already Known on This Subject?:**

- VHIs (e.g., telehealth, remote monitoring, digital health education) are promising solutions to address health inequities in rural and remote areas, where limited access to timely, high‐quality care contributes to higher mortality, greater chronic disease burden and increased reliance on emergency services compared to urban populations.
- Research users, including community members, healthcare providers and policymakers, are often underrepresented in the planning, conduct and implementation of VHI projects, limiting the incorporation of rural community‐voiced priorities, needs and perspectives and reducing the responsiveness of VHIs that are ultimately intended for their use.
- Research partnerships, where academic researchers collaborate with research users, are increasingly recognised as best practice for developing, implementing and evaluating VHI projects that generate relevant, meaningful innovations capable of promoting sustainable change in rural and remote communities.
- Building meaningful partnerships with rural and remote communities is challenging and time‐intensive; research may default to Western paradigms where researchers take dominant roles, which risks undermining trust and the long‐term impact of VHIs.

**What Does This Paper Add?:**

- The diverse set of partnership processes (principles, strategies) outlined in this review represents the ‘*how*’ of partnerships, offering a flexible menu of options that researchers and users can adapt to different contexts and study objectives to guide their engagement with RR communities.
- This review contributes to the literature by distinguishing Indigenous and non‐Indigenous partnership strategies and principles in rural VHI projects. Studies involving Indigenous partnerships often provide richer data and more detailed reporting on partnership processes.
- Partnership outcomes and impacts, *the* ‘*why*’, highlight the value, complexity and challenges of engagement, providing an evidence‐informed rationale for integrating partnerships into future research.
- This review offers practical guidance to support engagement across project planning, development and implementation, fostering early buy‐in, trust and sustained community participation.
- By outlining relationship‐centred processes grounded in shared principles and strategies, this review represents a step toward culturally safe, relevant and sustainable VHI in RR communities, where researchers work *with* and *for* research users.

## 1 Introduction

Research users, including community members, healthcare providers and policymakers, have traditionally been underrepresented in the design and implementation stages of VHI projects intended for their use. Consequently, these projects have often remained predominantly researcher‐oriented, lacking direct incorporation of community‐voiced challenges, perspectives and needs into project planning, conduct and implementation [1, 2]. Research partnerships, defined as collaborations between academic researchers and research users, are increasingly recognised as best practices for the development, implementation and evaluation of VHI projects designed for and with communities [3, 4, 5, 6]. The increasing emphasis on partnered approaches in project development is supported by accumulating evidence of their value in generating innovations that are relevant, meaningful and capable of promoting sustainable change [7].

VHI, including telehealth, remote monitoring and digital health education, represent promising solutions to address health inequities in rural and remote communities [8, 9]. Limited access to timely, high‐quality care in these communities contributes to higher mortality rates, a greater burden of chronic illness and increased reliance on emergency services compared to urban populations [10, 11, 12]. VHI can help bridge these gaps by expanding service availability, thus improving patient outcomes, addressing workforce shortages and reducing travel burden [13, 14, 15].

Meaningful engagement of those who can benefit from these innovations (i.e., research users) ensures that the perspectives and lived experiences of rural and remote communities are integrated into their design, evaluation and implementation, thus ensuring that the projects align with local needs and realities [16]. A partnership model grounded in shared decision‐making and knowledge co‐creation represents a shift toward more equitable systems and can support community‐centred adoption of high‐quality VHI in rural settings [17, 18, 19]. By fostering collaboration, researchers can better account for community‐specific contexts, including diverse cultures, preferences, needs and capacities [20, 21]. In turn, innovation projects can be tailored to align with local realities, enhancing their long‐term sustainability while strengthening community empowerment and capacity building [22, 23]. However, building meaningful and trusting partnerships with rural and remote communities presents unique challenges and requires time. Researchers may still default to dominant roles in decision‐making and implementation, reflecting conventional Western research paradigms [24, 25]. Such hierarchical dynamics risk misalignment with the diverse realities of rural and remote communities, eroding trust and undermining the long‐term impacts of VHI, particularly in geographically isolated settings [26].

Therefore, despite the recognised benefits of such research partnerships, there are gaps in our understanding of the most effective partnership processes capable of best supporting successful VHI projects. Given the relative nascency of such partnerships in VHI design, development and implementation, few systematic overviews have examined partnership principles and strategies, or evaluations of their effectiveness [18, 24, 27]. These gaps are further compounded by inconsistent transparency and reporting of research partnership approaches with research users [28].

Guided by the framework described by Hoekstra et al. [6], this scoping review provides a systematic overview of partnership processes (principles and strategies) and effects (outcomes/impacts) of rural and remote virtual health research partnerships. The findings inform our understanding of research partnerships in this area and the development of resources to support rural and remote VHI partnerships. Ultimately, by building capacity in VHI partnerships, the findings can contribute to the development of health innovation projects that are more meaningful, relevant and useful for rural and remote communities.

The research questions of this scoping review were as follows:

1. What research partnership principles and strategies have been reported to guide virtual health innovation projects in rural and remote communities?
2. What outcomes and impacts of partnership approaches in virtual health innovation projects have been reported?

## 2 Methods

### 2.1 Project Overview

This scoping review systematically mapped research partnerships in the context of rural and remote VHI projects. Specifically, we extracted and synthesised the principles, strategies, outcomes and/or impacts discussed in articles regarding VHI research partnerships. Our analysis was guided by a consensus‐based framework encompassing four key partnership domains: principles, strategies, outcomes and impacts [6].

This scoping review followed the steps outlined by Arksey and O'Malley [29], Levac et al. [30] and the Preferred Reporting Items for Scoping Reviews (PRISMA‐ScR) [31]. A pragmatic approach was taken, emphasising practical outcomes and the usefulness of knowledge within a specific context, with the primary objective of applying research to find solutions to practical problems in real‐world settings [32].

This project was conducted in a partnership, in which we meaningfully engaged research partners with diverse perspectives (C.C., A.S., Hybrid Care Research Collaborative (HCRC) members) throughout the review process. At inception, the lead author (F.H.) and trainees (V.T., J.Y., J.B.) held meetings with CC and AS to define the scope and inclusion criteria to maximise relevance. Ongoing engagement has been maintained through email updates and the sharing of key documents, including the study protocol, data extraction and analysis summaries, and manuscript drafts. All partners were invited to provide feedback on the relevance and application of the findings, the clarity and coherence of the categorisation, and the accessibility and appropriateness of the content and format. Initial findings were presented by co‐authors (V.T., J.Y.) to an interdisciplinary HCRC for both oral and written feedback and input. HCRC members were invited to complete a Qualtrics survey (https://osf.io/ua3wf) to provide structured input on identified gaps, future research directions and study limitations. This iterative feedback process promoted meaningful inclusion of diverse perspectives throughout the review and helped shape its interpretation, structure and contributions to both academic and non‐academic contexts. Details on partner names, affiliations and roles are provided in the Appendix S1, while research reflexivity details, including the positionality of the first and senior authors and community power dynamics, are provided in Appendix S2.

### 2.2 Search Strategy

An academic librarian (J.J.) searched five health databases (Medline, Embase, CINAHL, PsycINFO, Web of Science) from January 2020 to May 2024 and updated in December 2024. Databases were selected to ensure comprehensive, multidisciplinary coverage of relevant literature on rural and remote VHI research partnerships, including biomedical (MEDLINE), nursing and allied health (CINAHL), psychological (PsycINFO) and multidisciplinary health sciences (Scopus/Web of Science). The search strategy was informed by a previous review of reviews by the senior author, Hoekstra et al. [6], and developed in consultation with another academic librarian (M.V.) with expertise in searching for research partnership approaches. Our search strategies are available on OSF (https://osf.io/aszxh).

The search focused on primary studies published between 2020 and 2024. This timeline was chosen based on literature indicating increased prevalence of the development and implementation of VHI precipitated by the COVID‐19 pandemic, as well as evidence of efforts to apply these innovations in rural and remote communities [33, 34, 35]. Our search strategy consisted of three components:

1. Search terms
2. related to research partnerships (e.g., community‐engaged research, participatory research or co‐design).
3. Search terms
4. related to virtual health innovations (e.g., Telehealth, remote monitoring, mobile health apps and online education resources).
5. Search terms
6. related to rural, remote and Indigenous communities.

The academic librarian (J.J.) conducted the searches and imported the findings into Covidence, where duplicates were removed automatically.

### 2.3 Inclusion Criteria

We applied distinct inclusion criteria across two phases: title/abstract and full‐text screening. During abstract screening, we capture papers indicating research partnerships and VHI by applying broader, more inclusive criteria to minimise the risk of prematurely excluding relevant studies. Stricter criteria were applied during full‐text screening, including only articles explicitly describing partnership processes (strategies or principles) and/or partnership effects (outcomes or impacts). Table S3.1 provides a summary of the abstract/title screening criteria, with additional details and rationale available in Appendix S3.

Articles were included if they explicitly described, reflected and/or evaluated a research partnership or collaborative research activity in the development, evaluation and/or implementation of VHI in rural and/or remote communities. Rural or remote communities were defined as areas located outside population centres, including urban and metropolitan areas [36]. Eligible articles had to report at least one partnership principle, strategy or outcome/impact. We defined ‘principle’ as fundamental norms, rules or values of what is desirable and positive for a person, organisation or community that help to determine the rightfulness or wrongfulness of their actions. Strategies are concrete, observable actions to achieve a certain outcome. Outcomes/impacts are short‐ or long‐term effects resulting either directly or indirectly from the application of a research partnership approach [37]. Table 1 presents a condensed version of the full‐text criteria; the full version is available in Appendix S3 (Table S3.2).

**TABLE 1:** Condensed full‐text screening inclusion and exclusion criteria.

	Inclusion criteria	Exclusion criteria
Population	– Articles focusing on people living in (Indigenous) rural or remote communities and/or health professionals/administrators working in (Indigenous) rural or remote community settings.	– Articles focusing on people living in urban areas. – Articles focusing on people living or working in both urban and rural communities without a specific focus on people living in rural communities. – Articles focusing on urban Indigenous communities.
Intervention	– Articles describing the development, evaluation and/or implementation of a *virtual health innovation* with the aim of improving healthcare and services in a rural and/or remote community. – *Virtual health innovation* is defined as: ○ *Any technology, method or approach that leverages digital tools and platforms to improve delivery, accessibility, efficiency or quality of healthcare services remotely*. *Virtual health innovations include telemedicine platforms, mobile health apps, wearable devices, remote monitoring systems, virtual reality applications for healthcare training and therapy, artificial intelligence‐driven diagnostic tools and online health education resources* [11].	
Partnership	– Articles meeting our definition of *health research partnership*. ○ *Research partnership* is defined as: ○ *Individuals, groups or organisations engaged in collaborative research activity involving at least one researcher (e.g., individual affiliated with an academic institution) and any research user (e.g., decision or policy maker, health care administrator or leader, community agency, charities, network, patients etc.)* [37]. – Articles describing, reflecting and/or evaluating a *research partnership* OR describing, reflecting and/or evaluating at least one *collaborative research activity*. ○ *Collaborative research activity* is defined as: ○ *An activity or moment in the process of planning, conducting or disseminating research in which there is an indication of shared decision‐making between at least one researcher and at least one research user* [37].	– Articles not meeting our definition of *health research partnership* (e.g., physician‐patient partnership, student‐teacher partnership) or not providing enough information about the collaborative research activity to determine eligibility. – Articles related to patient engagement in health care decisions instead of research. – Articles where research users are *only* included as participants and not as research partners (e.g., Delphi studies). – Articles *describing* a research partnership approach or collaborative research activity without *reflecting* or *evaluating* it were excluded if: ○ *All* research users were also *all* participants in the research (100% overlap between participants and research users) OR ○ Research users were only engaged in the design of the study and not in the implementation or dissemination phase.
Outcomes	– Articles describing at least one clear example of a *principle* that was used or could be used to guide a research partnership team OR at least one clear example of a *strategy* used to engage research users in the collaborative research activity OR at least one clear example of an outcome/impact of the collaborative research activity.	– Articles that do not describe a clear example of a partnership principle, strategy and/or outcome/impact.

*Note:* Inclusion and exclusion criteria were structured using the PICO framework: Population (P), criteria requiring the involvement of specific research users in virtual health innovation research partnerships; Intervention (I), criteria related to the virtual health innovation itself; Comparison (C), not applicable here; Outcome (O), criteria for reported partnership processes and the effects of research partnerships on virtual health innovation.

### 2.4 Screening Process

Screening was performed using Covidence (Veritas Health Innovation, Melbourne, Australia. Available at www.covidence.org). Two screening pairs (J.B. & J.Y., E.T. & V.T.) reviewed each article independently based on the predefined criteria for both abstract and full‐text screening. Covidence automatically calculated interrater reliability for each screening pair during both phases. Throughout screening, we ensured the average Kappa score met the target of ≥ 0.6, calculated by the Covidence software. Disagreements were resolved through discussion between the screening pairs. If consensus could not be reached, the senior author (F.H.) was consulted.

### 2.5 Data Extraction and Analysis

Data extraction was guided by the established partnership framework by Hoekstra et al. [6] Data extraction occurred in two phases: general extraction of study and partnership characteristics; and extraction of partnership processes and effects. In the first phase, one team member (F.H., E.T., J.B., J.Y. or V.T.) conducted general extraction of study characteristics (e.g., bibliographic information, study design, aims), VHI characteristics (e.g., type, description, effectiveness), target population (e.g., rural, remote, Indigenous communities) and partnership characteristics (e.g., key terms, members, definitions, level of engagement) using an Excel spreadsheet. Team members consulted the senior author (F.H.) to resolve uncertainties (e.g., definitions, descriptions, study design), and the final version was cross‐validated between two reviewers (V.T. and J.Y.).

In the second phase, three team members (E.T., J.Y. and V.T.) applied directed qualitative content analysis [38] to extract partnership principles, strategies and outcomes using NVivo 14 (Lumivero, Denver, CO, USA). For data classification, we used an adapted codebook from a previous review [19] and a review of reviews [6]. Extractions were limited to the methods, results and discussion sections to ensure that principles, strategies and outcomes were explicitly reflected in the research process. To ensure consistency, the senior author (F.H.) independently extracted data from 10% of the articles (*n* = 6) and cross‐validated these with the respective team member. Outcomes and impacts were extracted only from studies reporting formal partnership evaluations, defined as those using quantitative (e.g., rating scales), qualitative (e.g., interviews, focus groups) or mixed methods approaches specifically designed to assess partnership processes or outcomes.

All codes and subcodes were transferred to Excel, with each article indicating at least one principle, strategy and outcome/impact coded as ‘1’. The findings were presented in a table and divided into two groups: Indigenous or non‐Indigenous partnership. Indigenous/non‐indigenous distinctions were made post hoc based on article content, not as a predefined eligibility criterion, to identify potential differences in the reported partnership principles between the two groups, such as the presence of principles uniquely relevant to Indigenous partnerships, recognising that Indigenous communities may have distinct cultural values, histories and priorities. The percentage of articles reporting principles and strategies within each classification was calculated to compare reporting frequencies between the two groups. Findings on outcomes and impacts were not categorised by Indigenous or non‐Indigenous partnerships due to the small sample size of formal evaluation studies. Detailed data extraction and analysis processes are available in Appendix S4.

## 3 Results

### 3.1 Literature Search

Before the removal of duplicate records, our search strategy identified 22 476 records. After automatic duplicate removal in Covidence, a total of 7413 unique records were retained for screening. Following title and abstract screening, 6880 articles were excluded, and the remaining 533 were subjected to full‐text review. One additional article, which was identified by independent reference searching, was included for full‐text review. A total of 85 articles were included in this scoping review. Interrater reliability between screeners was considered ‘moderate to substantial’ for both abstract screening (mean Cohen's Kappa for the four screening pairs: 0.93, 0.59, 0.60, 0.74; M = 0.72) and full‐text screening (mean Cohen's Kappa for the three screening pairs: 0.95, 0.93, 0.82; M = 0.90). The PRISMA 2020 flow diagram of the study selection process was presented as Supporting Information (Figure S5.1; Appendix S5). References from the included articles are presented in Appendix S6, and those that were excluded are available on OSF (https://osf.io/p2ntk). Table 2 provides an overview of the study characteristics of the 85 articles included. Additional details about the included studies are available on OSF (https://osf.io/ne243).

**TABLE 2:** Study characteristics.

First author	Year	Country	Title	Innovation category	Partnership information type			Partnership key term
					Desc	Refl	Eval	
*Non‐Indigenous* (*N* = 45)								
Bishop [39]	2023	Australia	Using integrated knowledge translation to address a rurally based time‐ critical knowledge gap during the COVID‐ 19 pandemic: a multimethods study in Victoria, Australia	Telehealth	Yes	Yes	Yes	IKT
Brown [40]	2020	USA	Description, utilization and results from a telehealth primary care weight management intervention for adults with obesity in South Carolina	Telehealth	Yes	No	No	Partnerships
Dahildahil [41]	2023	The Philippines	User‐centred design in time and resource‐limited settings: enhancing the usability of ‘Hearing for Life’ (*HeLe*) device	Telehealth	Yes	Yes	No	User‐centred design
Kirkland [42]	2023	USA	Dissemination of remote patient monitoring: an academic‐community primary care partnership in South Carolina	Telehealth	Yes	No	No	Academic‐community partnership
Klager [43]	2023	Australia	Optimising co‐design processes in telemedicine innovation‐developing a telemedical solution for emergency medical services	Telehealth	Yes	Yes	Yes	Co‐design
Mason [44]	2021	USA	Amplifying expertise in rural West Virginia through Project ECHO: impactful partnerships and community‐engagement	Telehealth	Yes	Yes	No	WV Project ECHO
Miyamot [45]	2021	USA	The Sexual Assault Forensic Examination Telehealth (SAFE‐T) Center: A comprehensive, nurse‐led telehealth model to address disparities in sexual assault care	Telehealth	Yes	No	No	Community‐engaged participatory approach
Ramanadhan [46]	2022	India	A model for sustainable, partnership‐based telehealth services in rural India: An early process evaluation from Tuver village, Gujarat	Telehealth	Yes	Yes	Yes	Business of Humanity Approach
Rahimipour Anaraki [47]	2022	Canada	Virtual healthcare in rural and remote settings: a qualitative study of Canadian rural family physicians' experiences during the COVID‐19 pandemic	Telehealth	Yes	No	No	Community‐based participatory approach
Rohan [48]	2022	USA	Pairing Project ECHO and patient navigation as an innovative approach to improving the health and wellness of cancer survivors in rural settings	Telehealth	Yes	No	No	Project ECHO partnership with participating clinics and health centres
Torres Diaz [49]	2021	USA	Implementation for Sustained Impact in Teleophthalmology (I‐SITE): applying the *NIATx Model* for tailored implementation of diabetic retinopathy screening in primary care	Telehealth	Yes	No	No	I‐SITE implementation team
Tsai [50]	2024	USA	Collaborative international partnership: enabling the development of a state‐of‐the‐art minimally invasive surgical centre in rural Uganda through telementoring	Telehealth	Yes	No	No	Collaboration
Woodward [52]	2024	USA	Transitioning an implementation research intervention to a sustained clinical service: telehealth primary care mental health integration implementation in Veterans Health Administration	Telehealth	Yes	No	No	Partnerships
Aronoff‐Spencer [53]	2022	USA	Designing a framework for remote cancer care through community co‐design: participatory development study	Mobile health tools	Yes	Yes	Yes	Human‐centred participatory design
Dabengwa [22]	2022	Zimbabwe	A participatory learning approach for the development of a maternal mobile health technology in Zimbabwe	Mobile health tools	Yes	No	No	Participatory learning approaches
Fowler [54]	2021	USA	Translating family‐based behavioural treatment for childhood obesity into a user‐friendly digital package for delivery to low‐income families through primary care partnerships: The MO‐CORD study	Mobile health tools	Yes	No	No	User‐centred design approach
Guerra [55]	2024	Columbia	Fostering collective action for adolescent well‐being: citizen science in a Colombian semi‐rural area	Mobile health tools	Yes	Yes	No	Citizen science
Kim [56]	2023	USA	The ACTIVATE digital health pilot programme for diabetes and hypertension in an underserved and rural community	Mobile health tools	Yes	Yes	No	Co‐design
Larson [57]	2020	USA	A contextual approach to inform a mobile health application for adolescent health	Mobile health tools	Yes	No	No	CBPR
Nkangu [58]	2024	Cameroon	The role of intersectoral collaboration and continuous stakeholder engagement in the implementation of the BornFyne PNMS project in Cameroon	Mobile health tools	Yes	Yes	No	Intersectoral collaboration
Obegu [59]	2023	Cameroon	Community participation for reproductive, maternal, newborn and child health: insights from the design and implementation of the BornFyne‐prenatal management system digital platform in Cameroon	Mobile health tools	Yes	Yes	No	Participatory research design
O'Donnel [60]	2024	USA	A web‐based peer support network to help care partners of people with serious illness: co‐design study	Mobile health tools	Yes	Yes	No	Co‐design framework
Pozuelo [21]	2023	USA	User‐centred design of a gamified mental health app for adolescents in Sub‐Saharan Africa: multicycle usability testing study	Mobile health tools	Yes	No	No	Co‐design
Sharma [61]	2024	USA	Development and beta test of a faith‐based Facebook intervention for smoking cessation in rural communities (FAITH‐CORE)	Mobile health tools	Yes	Yes	No	Community engagement
Veliz Reyes [62]	2024	UK	Codesign principles for the effective development of digital heritage extended reality systems for health interventions in rural communities	Mobile health tools	Yes	Yes	No	Co‐design
Wali [63]	2023	Canada	Bridging community and clinic through digital health: Community‐based adaptation of a mobile phone‐based heart failure programme for remote communities in Uganda	Mobile health tools	Yes	Yes	No	Co‐development
Bugnon [64]	2021	Switzerland	Improving primary care medication processes by using shared electronic medication plans in Switzerland: lessons learned from a participatory action research study	Education	Yes	Yes	No	PAR
Coury [65]	2024	USA	Engaging with rural communities for colorectal cancer screening outreach using modified Boot Camp Translation	Education	Yes	No	No	Collaborative community approach
Davis [66]	2022	Canada	Paving the way for electronic patient‐centred measurement in team‐based primary care: integrated knowledge translation approach	Education	Yes	No	No	IKT
Dindo [67]	2023	USA	Feasibility of delivering a virtual 1‐day acceptance and commitment therapy workshop to rural veterans through community partnerships	Education	Yes	Yes	No	Community‐engaged methods and non‐traditional treatment approaches
Gordon [68]	2023	USA	Research‐practice partnership: supporting rural cancer survivors in Montana	Education	Yes	No	No	RPP model
Heitkamp [69]	2021	USA	Use of evidence‐based technology to assist the behavioural health workforce: A case study on technology transfer systems	Education	Yes	No	No	Meaningful collaboration
Hinrichs‐Kinney [70]	2024	USA	Mixed‐method evaluation to understand clinician perspectives of a programme to implement high intensity resistance rehabilitation into skilled nursing facilities	Education	Yes	Yes	Yes	Multicomponent implementation programme
Hoffman [71]	2021	USA	Collaboration is key: implications for successful rural opioid misuse prevention programming	Education	Yes	Yes	No	Cooperative Extension's National Framework for Health and Wellness
Kohn [72]	2024	USA	Adapting evidence‐based falls prevention programmes for remote delivery—implementation insights through the RE‐AIM evaluation framework to promote health equity	Education	Yes	No	No	Collaboration
Lavender [73]	2024	USA	Building interprofessional educational bridges internationally: a reflection on our international partnership to equip future healthcare professionals with skills to care for rural and marginalised populations	Education	Yes	Yes	No	International partnership
Pfeuffer [74]	2022	Germany	Evaluation of a health information exchange system for geriatric health care in rural areas: development and technical acceptance study	Education	Yes	Yes	Yes	CBPR
Please [75]	2024	UK	Virtual reality technology for surgical learning: qualitative outcomes of the first virtual reality training course for emergency and essential surgery delivered by a UK‐Uganda partnership	Education	Yes	No	No	Collaboration
Porter [76]	2021	USA	A novel behavioural intervention for rural Appalachian cancer survivors (weSurvive): participatory development and proof‐of‐concept testing	Education	Yes	Yes	No	Participatory process
Sbaffi [77]	2023	UK	Promoting well‐being among informal caregivers of people with HIV/AIDS in rural Malawi: community‐based participatory research approach	Education	Yes	Yes	No	CBPR
Van Pelt [78]	2021	The Netherlands	The development of an electronic clinical decision and support system to improve the quality of antenatal care in rural Tanzania: lessons learned using intervention mapping	Education	Yes	Yes	Yes	Collaboration
Yelton [79]	2021	USA	‘Talk About Cancer and Build Healthy Communities’: how visuals are starting the conversation about breast cancer within African‐American communities	Education	Yes	Yes	No	Community‐engaged participatory approach
Aal [80]	2023	Switzerland	CareConnection – a digital caring community platform to overcome barriers of asking for, accepting and giving help	Cultural tailored programmes	Yes	Yes	No	PAR
Elk [81]	2020	USA	Developing and testing the feasibility of a culturally based tele‐palliative care consult based on the cultural values and preferences of southern, rural African American and White community members: a programme by and for the community	Cultural tailored programs	Yes	Yes	Yes	CBPR
Holst [82]	2021	Norway	Development of digital health messages for rural populations in Tanzania: multi‐ and interdisciplinary approach	Cultural tailored programmes	Yes	Yes	No	Multi‐ and interdisciplinary collaboration
*Indigenous* (*N* = 40)								
Beks [83]	2023	Australia	Implementation of telehealth primary health care services in a rural Aboriginal Community‐Controlled Health Organization during the COVID‐19 pandemic: a mixed‐methods study	Telehealth	Yes	No	No	Community‐based approach
Dunn [84]	2021	Canada	ECHO plus: improving access to hepatitis C care within Indigenous communities in Alberta, Canada	Telehealth	Yes	Yes	No	Collaboration and co‐design
Leader [85]	2023	Canada	‘Working on a Shoestring’: critical resource challenges and place‐based considerations for telehealth in northern Saskatchewan, Canada	Telehealth	Yes	No	No	Community‐based
Linnaranta [86]	2024	Canada	Views on a culturally safe psychotherapeutic treatment by Inuit in Quebec: co‐design of cognitive behavioural therapy manual and virtual exposure environments	Telehealth	Yes	Yes	No	Co‐design
Mah [87]	2024	Canada	Relational work is the work: virtual healthcare transformation for rural, remote and First Nation Communities in British Columbia	Telehealth	Yes	Yes	No	RTVS collaborative relationships
McIlduff [88]	2022	Canada	Engaging Indigenous older adults with technology use to respond to health and well‐being concerns and needs	Telehealth	Yes	Yes	No	CBPR
Novak Lauscher [89]	2023	Canada	Real‐time virtual support as an emergency department strategy for rural, remote and indigenous Communities in British Columbia: descriptive case study	Telehealth	Yes	Yes	No	Interorganisational collaboration
Novak Lauscher [90]	2023	Canada	Real‐time virtual supports improving health equity and access in British Columbia	Telehealth	Yes	No	No	Partnership Pentagram Plus
Roach [91]	2022	Canada	Access, Relationships, Quality and Safety (ARQS): a qualitative study to develop an Indigenous‐centred understanding of virtual care quality	Telehealth	Yes	Yes	No	PAR
Roach [92]	2023	Canada	Access, Relationships, Quality and Safety (ARQS): a qualitative study to cocreate an Indigenous patient experience tool for virtual primary care	Telehealth	Yes	No	No	Patient‐oriented research
Robler Kleindienst [93]	2020	USA	Hearing Norton Sound: community involvement in the design of a mixed methods community randomised trial in 15 Alaska Native communities	Telehealth	Yes	No	No	Community randomised trials
Ross [94]	2024	USA	Impact of tele‐antimicrobial stewardship at two small community hospitals in partnership with an academic medical centre: two years of experience	Telehealth	Yes	No	No	Academic medical centre partnership
Young [95]	2024	Canada	Ohpikihawasowin (grounding and guiding on the path to be a healthy parent): virtual adaptation of an Elders mentoring programme to support maternal and child wellbeing during the COVID‐19 pandemic	Telehealth	Yes	No	No	CBPR
Batai [96]	2022	USA	Formative assessment to improve cancer screenings in American Indian men: Native Patient Navigator and mHealth texting	Mobile health tools	Yes	Yes	No	CBPR
Bennett‐Levy [97]	2021	Australia	From digital mental health to digital social and emotional wellbeing: how Indigenous community‐based participatory research influenced the Australian Government's digital mental health agenda	Mobile health tools	Yes	Yes	No	CBPR
Britt [98]	2021	USA	‘Sharing Hope and Healing’: a culturally tailored social media campaign to promote living kidney donation and transplantation among Native Americans	Mobile health tools	Yes	Yes	No	CBPR
Glennie [99]	2022	Australia	Engaging remote Aboriginal communities in COVID‐19 public health messaging via crowdsourcing	Mobile health tools	Yes	No	No	Participatory approach
Akearok [ 100]	2020	Canada	Cultural orientation and safety app for new and short‐term health care providers in Nunavut	Mobile health tools	Yes	No	No	Partnership
Henson [101]	2022	Australia	Mature aged Aboriginal and Torres Strait Islander adults are using digital health technologies (original research)	Mobile health tools	Yes	No	No	Co‐design research
Henson [102]	2023	Australia	Amplifying older Aboriginal and Torres Strait Islander women's perspective to promote digital health equity: co‐designed qualitative study	Mobile health tools	Yes	No	No	Rambaldini Model of Collective Impact
Henson [103]	2024	Australia	Wearables are a viable digital health tool for older Indigenous adults living remotely in Australia (research)	Mobile health tools	Yes	No	No	Co‐design
Jacklin [104]	2020	USA	Peace of mind: A community‐industry‐academic partnership to adapt dementia technology for Anishinaabe communities on Manitoulin Island	Mobile health tools	Yes	No	No	CBPR
Petrie [105]	2024	Canada	Bringing care close to home: remote management of heart failure in partnership with Indigenous communities in Northern Ontario, Canada	Mobile health tools	Yes	No	No	Collaborative model of care
Roh [106]	2023	USA	Mobile web app intervention to promote breast cancer screening among American Indian women in the Northern Plains: feasibility and efficacy study	Mobile health tools	Yes	No	No	CBPR
Vigil‐Hayes [107]	2021	USA	Integrating cultural relevance into a behavioural mHealth intervention for Native American youth	Mobile health tools	Yes	Yes	No	CBPR
Binks [16]	2024	Australia	Adapting and translating the ‘Hep B Story’ App the right way: a transferable toolkit to develop health resources with, and for, Aboriginal people	Education	Yes	Yes	No	PAR
Blind [17]	2023	USA	Training Indigenous community researchers for community‐based participatory ethnographic dementia research: a second‐generation model	Education	Yes	Yes	No	Community‐based participatory research
Cueva [108]	2019	USA	An evaluation of cancer education webinars in Alaska	Education	Yes	Yes	No	Community‐based participatory action
Devan [109]	2021	New Zealand	‘A coalition of the willing’: experiences of co‐designing an online pain management programme (iSelf‐help) for people with persistent pain	Education	Yes	Yes	Yes	Co‐design, PAR
Kerrigan [110]	2023	Australia	Countering the ‘wrong story’: a Participatory Action Research approach to developing COVID‐19 vaccine information videos with First Nations leaders in Australia	Education	Yes	Yes	No	PAR
Nixon [111]	2024	Australia	Healthcare social network research and the ECHO model: Exploring a community of practice to support cultural brokers and transfer cultural knowledge	Education	Yes	Yes	No	Collaborative partnerships
Peake [112]	2021	Australia	Meaningful engagement with Aboriginal communities using participatory action research to develop culturally appropriate health resources	Education	Yes	Yes	No	PAR with the research topic Yarning (RTY) framework
Perry [113]	2022	New Zealand	iSelf‐Help: a co‐designed, culturally appropriate, online pain management programme in Aotearoa	Education	Yes	Yes	No	PAR
Katapally [114]	2020	Canada	Smart Indigenous Youth: the smart platform policy solution for systems integration to address Indigenous youth mental health	Education	Yes	Yes	No	CBPR
Teufel‐Shone [115]	2022	USA	Maintaining the partnership between a tribal breast and cervical cancer programme and a university‐based cancer prevention centre during COVID‐19 lock‐down restrictions‐a case study	Education	Yes	Yes	No	NCAP and NNBCCPP collaboration
Acharibasam [116]	2022	Canada	Exploring health and wellness with First Nations communities at the ‘Knowing Your Health Symposium’	Cultural tailored programmes	Yes	Yes	No	CBPR
Povey [117]	2022	Australia	Determining priorities in the Aboriginal and Islander Mental Health Initiative for Youth app second phase participatory design project: qualitative study and narrative literature review	Cultural tailored programmes	Yes	Yes	Yes	Participatory design approach
Rieger [118]	2021	Canada	Digital storytelling as a patient engagement and research approach with First Nations women: how the Medicine Wheel guided our *Debwewin* journey	Cultural tailored programmes	Yes	Yes	No	Collaborative engagement with patient partner
Sauvé [119]	2022	Canada	Stand Up for Indigenous Health: a simulation to educate residents about the social determinants of health faced by Indigenous Peoples in Canada	Cultural tailored programmes	Yes	No	No	Community‐based research
Snijder [120]	2021	Australia	Strong and Deadly Futures: co‐development of a web‐based wellbeing and substance use prevention programme for Aboriginal and Torres Strait Islander and non‐Aboriginal adolescents	Cultural tailored programmes	Yes	No	No	Participatory research

Abbreviations: Descr, the paper includes a description of the partnership approach; Eval, the paper includes an evaluation of the partnership approach. CBPR, community‐based participatory research; IKT, integrated knowledge translation; PAR, participatory action research; Refl, the paper includes a reflection of the partnership approach; RPP, research‐practice partnership; RTVS, real‐time virtual support.

### 3.2 Study and Partnership Characteristics

Most of the included articles (*n* = 41, 48.2%) were published between 2023 and 2024, followed by 2021 and 2022 (*n* = 36, 42.4%), with the remainder published between 2019 and 2020 (*n* = 8, 9.4%). The partnerships reflected geographically diverse representation, including studies from the United States (*n* = 37), Canada (*n* = 19), Australia (*n* = 12), the United Kingdom (*n* = 3), New Zealand (*n* = 2), Switzerland (*n* = 2), Cameroon (*n* = 2), Zimbabwe (*n* = 1), Norway (*n* = 1), the Netherlands (*n* = 1), Germany (*n* = 1), Austria (*n* = 1), India (*n* = 1), the Philippines (*n* = 1) and Colombia (*n* = 1).

Regarding the level of reporting on the partnership approach, 36 articles (48%) described but did not reflect on or evaluate the partnership approach or collaborative research activities. Another 40 articles (42%) both described and reflected on collaborative research activities, while 9 articles (10%) included a formal evaluation using either qualitative or quantitative methods.

### 3.3 Partnership Principles

We extracted 149 unique principles from 78 articles (OSF: https://osf.io/n349x), while the remaining seven articles did not report any principles. Most articles did not specify details of how or what partnership principles were used to plan, conduct and/or disseminate their VHI project. The principles were synthesised into 18 overarching principles, 2 of which were specific to research partnerships involving Indigenous communities. Table 3 provides an overview of the 18 overarching principles and the frequencies with which they were identified in articles describing non‐Indigenous and Indigenous research partnerships. Overall, articles discussing Indigenous research partnerships included more information on partnership principles compared to those describing non‐Indigenous research partnerships.

**TABLE 3:** Systematic overview of partnership principles.

Categories	Overarching principles	Context	
		Non‐Indigenous, *N* = 39	Indigenous, *N* = 39
Relationship building	Fostering respectful, trusting and sustainable relationships as the foundation for meaningful partnerships	18/39 (46.2%)	29/39 (74.4%)
Diverse expertise and knowledge	Fostering a respectful partnership that values diverse perspectives, local values and community expertise	5/39 (12.8%)	18/39 (46.2%)
Shared decision‐making	Promoting a participatory and community‐driven partnership that emphasises shared decision‐making, resource sharing and collaborative innovation	15/39 (38.5%)	29/39 (74.4%)
Address power dynamics and power sharing	Promoting an equitable partnership that addresses power imbalances, prioritises marginalised voices and mutual respect	8/39 (20.5%)	13/39 (33.3%)
Representation and inclusivity	Fostering an inclusive environment that prioritises and accurately represents the voices and experiences of all partners	6/39 (15.4%)	14/39 (35.9%)
Knowledge co‐production	Ensuring active and equal participation of all partners throughout the entire research process, from innovation development to dissemination.	17/39 (43.6%)	25/39 (64.1%)
Shared ownership	Prioritising shared ownership of research process and outcomes with research users	7/39 (17.9%)	11/39 (28.2%)
Knowledge translation and implementation	Cultivating a collaborative, holistic and iterative process to support sustainable knowledge integration, innovation implementation and social change.	17/39 (43.6%)	12/39 (30.8%)
Reflection and planning engagement approach	Fostering equitable, reflective and collaborative partnerships for meaningful impact	20/39 (51.3%)	21/39 (53.8%)
Flexibility in engagement approach	Adopting a flexible and context‐sensitive approach that balances standardised methods with tailored adaptations to meet partners' needs	1/39 (2.6%)	4/39 (10.3%)
Mutual benefit	Fostering culturally safe, ethical research practices that ensure mutual benefits for all partners involved.	4/39 (10.3%)	6/39 (15.4%)
Ensuring relevance of research	Ensuring that research and innovation are culturally respectful, inclusive and responsive to the needs and aspirations of all partners, with a particular focus on Indigenous priorities	15/39 (38.5%)	11/39 (28.2%)
Capacity building	Leveraging community strengths, leadership and resources to support capacity building, skill development and foster pride and a sense of community among research users.	8/39 (20.5%)	12/39 (30.8%)
Co‐learning	Promoting a reciprocal learning environment where knowledge is mutually shared, and learning is fostered between researchers and their partners	4/39 (10.3%)	8/39 (20.5%)
Communication between members	Fostering open, respectful and continuous communication to build and maintain effective partnerships	6/39 (15.4%)	8/39 (20.5%)
Ethical and practical issue	Commitment to ethical, inclusive and respectful engagement with partner communities, fostering safe and collaborative spaces that honour diverse knowledge systems and uphold relational accountability	9/39 (23.1%)	7/39 (17.9)
Respecting Indigenous culture, history and value	Acknowledge and integrate Indigenous histories, cultures and knowledge systems, recognising their impact on well‐being and the importance of ethical, inclusive and respectful engagement.	1/39 (2.6%)	14/39 (35.9%)
Cultural safety, sensitivity and relevance	Commitment to culturally sensitive and responsive innovation, guided by humility and respect for diverse communities	1/39 (2.6%)	20/39 (51.3%)

The most frequently identified principles were related to the following overarching principles:

1. Fostering respectful, trusting, collaborative and sustainable relationships as the foundation for meaningful and effective partnerships (47 of 85 articles, 55.3%).
2. Promoting a participatory and community‐driven partnership emphasising shared decision‐making, resource sharing and collaborative innovation (44 of 85 articles, 51.2%).
3. Ensuring active and equal participation of those most impacted throughout the entire research process, from co‐creation to dissemination and innovation development (42 of 85 articles, 49.4%).

The article with the greatest number of identified research partnership principles was by Blind et al. [17]. This community‐based participatory research (CBPR) project described and discussed the development of an online training programme designed to support Indigenous communities in the United States and Canada in conducting multisite ethnographic research on dementia. The coding manual is available on OSF (https://osf.io/8dy6f), and the unique principle codes identified in each included study can also be accessed (https://osf.io/qfs9n).

### 3.4 Partnership Strategies

We identified 80 partnership strategies from the 85 articles included in the analysis (OSF: https://osf.io/bjg5u). After merging strategy codes with similar meanings, we categorised 36 strategies as phase‐specific within the research process and 44 strategies as applicable across all phases. The strategies applicable throughout the research process were grouped into the following categories:

- Resources and Time
- Communication Activities and Methods
- Fostering Collaboration and Communication Processes
- Development of Norms, Rules and Expectations
- Partnership Initiation and Representation
- Education and Training

Although Indigenous and non‐Indigenous partnerships reported similar proportions of strategies, articles on Indigenous partnerships covered nearly all strategies applicable throughout research phases and included several unique ones. For instance, hosting gatherings in a sharing circle or outlining how to respect the Seven Grandfather Teachings were strategies reported only in Indigenous studies. This pattern suggests that certain strategies may be specifically tailored to, or more strongly emphasised within, Indigenous research contexts.

Across all partnerships, the most commonly reported strategies applicable across phases related to partnership initiation and representation, followed by communication activities and methods. Within initiation and representation, frequently reported strategies included establishing diverse teams of researchers and research users across sectors, roles and disciplines (*n* = 48/85, 56.5%) and establishing new partnerships or building upon existing ones (*n* = 45/85, 52.9%). Among communication‐related strategies, the most common was holding structured meetings—face‐to‐face, by phone or via conference calls (*n* = 45/85, 52.9%).

For phase‐specific strategies, most were identified in the planning phase, with involving research users in developing, evaluating and refining engagement processes and materials being most frequent (*n* = 28/85, 32.9%). In the conduct phase, the most reported strategy was involving research users in data analysis to ensure findings were relevant and accurate (*n* = 46/85, 54.1%). In the dissemination phase, the most common strategy was engaging partners to assist with developing tools and resources to share findings (*n* = 18/85, 21.2%). Tables 4 and 5 provide overviews of strategies across and within phases. Drafts of the partnership strategies coding manual and the unique strategy codes are publicly available on OSF: https://osf.io/autp3 and https://osf.io/r5ah7.

**TABLE 4:** Overview of partnership strategies applicable throughout the research process.

Categories	Example strategies	Context	
		Non‐Indigenous	Indigenous
Research and time	Apply for funding for collaborative research activities	7/46 (15.2%)	5/39 (12.8%)
	Compensate research users for their time	6/46 (13.0%)	3/39 (6.5%)
	Offer honorariums to research users for their time	2/46 (4.4%)	2/39 (5.1%)
Communication activities and methods	Use different tools (e.g., flipcharts, communication aids, interactive tools) to support research users' understanding and promote engagement.	9/46 (19.6%)	2/39 (5.1%)
	Generate information via interviews or consultations	5/46 (10.9%)	6/39 (15.4%)
	Generate information via focus groups, workshops or brainstorming sessions with research users	9/46 (19.6%)	4/39 (10.3%)
	Generate information via surveys or email	6/46 (13.0%)	5/39 (12.8%)
	Generate information via visual or active methods	5/46 (10.9%)	4/39 (10.3%)
	Utilise online platforms or web portals for research users' interaction	8/46 (17.4%)	9/39 (23.1%)
	Speak a common language between researchers and research users and tailor communication to the community context (e.g., in‐person oral communication aligned with local traditions).	0/46 (0%)	3/39 (7.7%)
	Hold structured meetings (face‐to‐face, phone, conference calls)	27/46 (58.7%)	18/39 (46.2%)
	Use consensus meetings (e.g., Nominal Group Technique)	2/46 (4.4%)	2/39 (5.1%)
Fostering collaboration and communication processes	Outline communication strategies at the onset of the partnership	0/46 (0%)	1/39 (2.6%)
	Maintain continuous dialogue and communication	13/46 (28.3%)	5/39 (12.8%)
	Establish community or patient advisory group (or committee)	6/46 (13.0%)	10/39 (25.6%)
	Engage consultants, moderators, local champions or liaisons to coordinate research users' engagement and support project oversight.	15/46 (32.6%)	10/39 (25.6%)
	Hold informal meetings	6/46 (13.0%)	1/39 (2.6%)
	Schedule meetings at times and locations convenient for research users, using communication and pre‐planning	4/46 (8.7%)	1/39 (2.6%)
	Apply the Two‐Eyed Seeing perspective	1/46 (2.2%)	4/39 (10.3%)
	Host gatherings in a sharing circle	0/46 (0%)	1/39 (2.6%)
Development of norms, rules and expectations	Develop shared goals and a common mission or agenda	4/46 (8.7%)	5/39 (12.8%)
	Develop and maintain agreed‐upon norms, rules and expectations	5/46 (10.9%)	6/39 (15.39%)
	Define research users' commitment, influence, readiness and engagement	4/46 (8.7%)	1/39 (2.6%)
	Outline how to respect the Seven Grandfather Teachings	0/46 (0%)	1/39 (2.6%)
	Start meetings with opening ceremony, rapport building or prayer	1/46 (2.2%)	3/39 (7.7%)
Partnership initiation and representation	Use a targeted strategy was used to identify and recruit research users (e.g., researcher sent out an email about the research idea)	15/46 (32.6%)	9/39 (23.1%)
	Establish or recruit advisory committee members from the local community (e.g., health educators, local leaders or community groups).	5/46 (10.9%)	3/39 (7.7%)
	Reach out and engage community organisations	3/46 (6.5%)	2/39 (5.1%)
	Establish a diverse team of researchers and engage diverse research users (selected across disciplines, sectors, roles and backgrounds) to ensure inclusivity and relevance in the research process.	24/46 (52.2%)	24/39 (61.5%)
	Expand the Community Advisory Board	3/46 (6.5%)	1/39 (2.6%)
	Establish new research partnerships and/or build upon existing ones (e.g., partnerships within an established network)	26/46 (56.5%)	19/39 (48.7%)
	Reach out to relevant communities (by researchers)	4/46 (8.7%)	5/39 (12.8%)
	Recruit research users and researchers via professionals' network or community network	10/46 (21.7%)	4/39 (10.3%)
	Establish an Aboriginal Governance Group	0/46 (0%)	2/39 (5.1%)
	Establish an Indigenous Advisory Group	0/46 (0%)	6/39 (15.4%)
	Involve Aboriginal Trainee in partnership initiation	0/46 (0%)	1/39 (2.6%)
	Involve Elder Representative in partnership initiation	0/46 (0%)	9/39 (23.1%)
Education and training	Educate and inform research users and researchers to build relevant knowledge and skills	13/46 (28.3%)	5/39 (12.8%)
	Educate or train research users in research methods	2/46 (4.4%)	1/39 (2.6%)
	Train researchers and research users to facilitate focus groups, including technical setup and handling challenging situations, to foster meaningful engagement	5/46 (10.9%)	1/39 (2.6%)
	Train research users to effectively share their perspectives and foster respectful, inclusive engagement	3/46 (7.7%)	1/39 (2.6%)
	Train research users and community partners on their roles and responsibilities to support future programme delivery	3/46 (6.5%)	1/39 (2.6%)
	Provide ongoing, tailored mentorship or training for researchers and research users	3/46 (6.5%)	0/39 (0%)
	Provide discipline specific training for researchers and research users and include opportunities to demonstrate their knowledge and skills.	17/46 (37.0%)	7/39 (17.9%)

**TABLE 5:** Overview of research process phase‐specific partnership strategies.

Research stage	Examples strategies	Context	
		Non‐Indigenous	Indigenous
Planning research	Co‐design the research process	2/46 (4.3%)	4/39 (10.3%)
	Co‐design the innovation with research users (e.g., leadership figure, community member, health care provider)	18/46 (39.1%)	8/39 (20.5%)
	Engage research users in planning the research process or implementation plan	15/46 (32.6%)	8/39 (20.5%)
	Involve research users in identifying and refining research question	5/46 (10.9%)	1/39 (2.6%)
	Identify relevant topics and key objectives	7/46 (15.2%)	4/39 (10.3%)
	Provide input to ensure cultural appropriateness of both the research planning and the development of related materials and interventions (e.g., intervention design, tools or resources).	8/46 (17.4%)	11/39 (28.2%)
	Provide input on the relevance, and inclusivity of the research and research materials.	5/46 (10.9%)	6/39 (15.4%)
	Provide recommendations to the study design	5/46 (10.9%)	11/39 (28.2%)
	Provide verbal approval before starting recruitment	1/46 (2.2%)	1/39 (2.6%)
	Involve research users in developing the study objectives, proposal, protocol	6/46 (13.0%)	8/39 (20.5%)
	Hold an initial meeting to build on prior knowledge and reach common ground.	2/46 (4.3%)	0/39 (0%)
	Involve research users in identifying potential project barriers, community needs and/or common goals.	5/46 (10.9%)	3/39 (7.7%)
	Identify the roles and contributions of research users and community partners at the outset.	3/46 (6.5%)	3/39 (7.7%)
	Involve research users in developing, evaluating and refining of research engagement process and materials	17/46 (37.0%)	11/39 (28.2%)
	Engage the advisory group and community members in the Conducting Phase	2/46 (4.3%)	3/39 (7.7%)
Conducting research	Assist with data collection and development of data management strategies	4/46 (8.7%)	4/39 (10.3%)
	Conduct Interviews	7/46 (15.2%)	8/39 (20.5%)
	Involve research users in focus group data collection (e.g., facilitation of focus groups, interviewing of focus groups)	11/46 (23.9%)	9/39 (23.1%)
	Translate focus group findings into recommendations for future research designs (by research users)	3/46 (6.5%)	1/39 (2.6%)
	Involve research users in data analysis to ensure the findings are relevant and accurate	13/46 (28.3%)	13/39 (33.3%)
	Review Literature (e.g., research users conducted literature review)	4/46 (8.7%)	2/39 (5.1%)
	Update research users (e.g., advisory board) on the research progress and results to gather feedback and facilitate discussion of findings.	8/46 (17.4%)	6/39 (15.4%)
	Regularly Monitor research users' engagement using check‐ins and knowledge check	1/46 (2.2%)	0/39 (0%)
	Use Indigenous research methodologies to engage communities	2/46 (4.3%)	6/39 (15.4%)
Disseminating research	Engage Traditional Knowledge Keepers in conducting research	1/46 (2.2%)	0/39 (0%)
	Advise, formulate and initiate action plan	2/46 (4.3%)	2/39 (5.1%)
	Approve the project through the advisory group	0/46 (0%)	3/39 (7.7%)
	Co‐author research outputs	1/46 (2.2%)	5/39 (12.8%)
	Develop key messages	2/46 (4.3%)	1/39 (2.6%)
	Involve research users in knowledge translation activities and development of dissemination strategies	7/46 (15.2%)	4/39 (10.3%)
	Assist with communicating and disseminating the findings to other research users (by the researchers).	5/46 (10.9%)	4/39 (10.3%)
	Review research materials and findings by researchers.	1/46 (2.2%)	3/39 (7.7%)
	Assist with developing tools and resources to disseminate findings	7/46 (15.2%)	11/39 (28.2%)
	Providing recommendation on how to adapt and tailor the innovation	7/46 (15.2%)	3/39 (7.7%)
	Involve research users in evaluating the innovation and provide feedback before finalising or scaling up the project.	9/46 (19.6%)	3/39 (7.7%)
	Assist with writing reports or scientific papers (by researchers)	0/46 (0%)	1/39 (2.6%)

### 3.5 Partnership Outcomes/Impacts

Our analysis identified 36 unique outcomes and impacts from 9 of the 85 papers that included partnership evaluation. Of these, 7 were non‐Indigenous partnerships and 2 involved partnerships with Indigenous communities. We classified outcomes/impacts into five major categories:

- Outcomes/impacts on researchers conducting partnership research (individual‐level)
- Outcomes/impacts on research users conducting partnership research (individual‐level)
- Outcomes/impacts on the relationship between researchers and research users (partnership‐level)
- Outcomes/impacts on the community or society
- Outcomes/impacts on the research process

Overall, 81% of the identified outcomes and impacts were classified as positive. The most frequently reported benefits were improved researcher understanding of the study area (56%) and increased community trust in research (56%). The most common challenges were additional time and financial burden on researchers (33%) and burden on research users (22%). The study by Devan et al. [108] identified the greatest number of different outcomes and impacts (*n* = 19), detailing the co‐design of an online pain management programme (iSelf‐help) for people with persistent pain, particularly within the Māori Indigenous population of New Zealand. Table 6 summarised the outcomes/impacts extracted from the nine articles that included formal partnership evaluation. The full data extraction table for partnership outcomes/impacts can be accessed on OSF (https://osf.io/zf6ab).

**TABLE 6:** Systematic overview of the partnership outcomes/impacts.

Outcomes or impacts on	Examples	% Papers
Researchers conducting partnership research (individual‐level)	Increased awareness of community issues	3/9 (33%)
	Researchers gained a better understanding of the area under study	5/9 (56%)
	Learned unmet needs of the community	2/9 (22%)
	Additional time and financial burden on researchers	3/9 (33%)
Research users involved in research partnerships (individual‐level)	Increased awareness of application of research, needs and resources	1/9 (11%)
	Research users' trust in research and researchers improved	2/9 (22%)
	Research users felt empowered	2/9 (22%)
	Research users felt listened to and valued	1/9 (11%)
	Research users had increased confidence and self‐worth after involvement	1/9 (11%)
	Research users learned to deal with their condition or condition of their friend/family member	1/9 (11%)
	Research users felt increased sense of accomplishment	3/9 (33%)
	Extended research users' social and support network	1/9 (11%)
	Improved access to information on treatment/management of illness	1/9 (11%)
	Research users felt a lack of meaningful involvement	1/9 (11%)
	Research users felt they were not being listened to	1/9 (11%)
	Research users felt frustrated	1/9 (11%)
	Research users felt dissatisfied/frustrated about formal research processes	2/9 (22%)
	Burden on research users	2/9 (22%)
Relationships between researchers and research users (partnership‐level)	Created mutual respect between researchers and users	2/9 (22%)
	Enhanced mutual understanding of language, work style, needs and constraints	4/9 (44%)
	Strengthened relationships, trust and goodwill	2/9 (22%)
	Greater partnership synergy	1/9 (11%)
	Increase in participants' trust and safety	2/9 (22%)
	Enhanced mutual learning of researcher and research users	1/9 (11%)
	May lead to conflict between researchers and partners	1/9 (11%)
Community or society	Improved or new community services and service delivery	2/9 (22%)
	Improved health and behavioural related outcomes for community	1/9 (11%)
	Created capacity to sustain the project in the community after funded time period/sustainable interventions	3/9 (33%)
	Increased community empowerment	1/9 (11%)
	Increased acceptability and trust of the research in the community	56%
Research process	Led to effective interventions/research	33%
	Unearthed new information	11%
	Created capacity to conduct and disseminate the research	11%
	Enhanced dissemination of research findings/knowledge translation	11%
	A more effective translation from research to practice	11%
	May lead to tokenism	11%

## 4 Discussion

This scoping review provided systematic overviews of diverse research partnership processes and effects reported in the literature on VHI projects aimed at improving access to quality healthcare services in rural and remote communities. The included studies originated from various countries, with the majority conducted in the United States, Canada and Australia.

Research partnerships have been applied to support a broad range of VHI projects, including research and quality improvement initiatives. The large number of digital health tools developed, implemented and/or evaluated within these projects reflects the growing acceptance of VHI as both viable alternatives and complements to in‐person care—a shift accelerated by the COVID‐19 pandemic, which necessitated the rapid adoption and adaptation of virtual innovations [121, 122]. This trend may also reflect the increasing adoption of VHI as feasible solutions to reduce healthcare disparities in communities, particularly by improving access and quality of care [123].

Nearly half of the identified studies included partnerships with Indigenous communities, aligning with literature emphasising the importance of partnership‐ or engagement‐based research in these contexts [21, 124, 125]. Such collaborative partnerships can incorporate Indigenous worldviews, ways of knowing and lived experiences as a foundation for meaningful engagement with Indigenous communities, which in turn supports the development of virtual health solutions that are more culturally relevant and safe [124–126]. Furthermore, the application of research partnerships to support a variety of rural VHI projects across continents, communities and types of digital health tools reflected in this review reinforced their implementation as a promising approach to enhancing the success, relevance and sustainability of these innovations at the community level [l6, 127, 128].

### 4.1 Partnership Processes

A variety of partnership processes were identified in rural and remote VHI projects. The most frequently reported principles and strategies were consistent with the broader literature, including a comprehensive review of reviews on partnership approaches across diverse research contexts and a prior review of spinal cord injury research [6, 19]. This may suggest that partnership processes are grounded in common foundations with broad applicability beyond rural and remote VHI. The identified principles may reflect consistent values and norms that researchers and research users regard as positive, whereas the strategies outline common actions for building and sustaining partnerships, such as engaging diverse research users to ensure inclusivity, maintaining regular communication, co‐developing norms and expectations, and providing training to build capacity.

We found that those describing partnership with Indigenous communities placed a stronger emphasis on relationality and community‐driven approaches, reflected in principles of shared decision‐making, relationship‐building and the co‐creation of knowledge and innovation. Two overarching principles were unique to Indigenous partnerships: respect for Indigenous culture, history and worldviews, and a commitment to cultural safety and culturally appropriate engagement. These findings resonate with the Cornerstones of Partnership Model of ethical Indigenous research, which emphasises Respect, Collaboration, Active Participation and Meeting Needs, and with the ethical research criteria of the ‘Four Rs’ (i.e., respect, relevance, reciprocity and responsibility) as the basis for culturally safe and meaningful collaboration [129, 131]. In line with these principles, strategies unique to Indigenous partnerships included establishing governance or advisory groups, involving Elders as representatives, and hosting gatherings in sharing circles, which facilitated the integration and exchange of Indigenous knowledge and culture within research engagement and decision‐making [129, 131].

Across different phases, Indigenous partnerships more often described strategies such as co‐designing research processes during planning, integrating Indigenous methodologies during conduct, and working with Indigenous partners to review research outputs and co‐author findings during dissemination. These strategies are consistent with recommendations that Indigenous partnerships centre on community needs and priorities through community‐based orientations and flexible, reflexive and participatory methods [129, 130, 132]. These processes likely reflect the frequent use of CBPR and PAR, which have been noted as suitable for Indigenous contexts and promising for addressing health disparities [128, 131, 132].

Non‐Indigenous partnerships, by contrast, more commonly emphasised principles related to reflective practice and deliberate planning within engagement processes, as well as the use of IKT approach. This aligns with our observation that non‐Indigenous partnerships tend to place greater emphasis on enhancing the relevance and practical utility of the VHI developed [133]. This interpretation is supported by literature indicating that rural and remote community members regard relevance as a key aspect of partnership engagement [134]. Therefore, teams collaborating with non‐Indigenous rural and remote communities may benefit from deliberate planning to facilitate equitable participation and ongoing reflection, together with a strong emphasis on knowledge exchange and translation. Such practices can help orient projects toward more actionable and sustainable change within the communities [131, 135].

### 4.2 Partnership Effects

Our review found that partnership outcomes and impacts in rural and remote VHI projects were predominantly positive—with 81% of studies reporting beneficial effects, consistent with the previous review [6]—which reported 68% positive outcomes. A particularly important outcome was increased community acceptance and trust in research, which is critical in Indigenous rural and remote communities, where historical injustices and scepticism toward externally led, academic‐driven initiatives persist [136–138].

Our review also highlighted challenging outcomes of research partnerships, particularly the increased time and resource burdens on both researchers and community partners—aligning with concerns raised in the literature [139]. However, no study explicitly analysed how partnership principles or strategies are linked to outcomes, making it challenging to determine which specific processes contribute to reported challenges or benefits. Furthermore, only 9 of the 85 studies formally evaluated partnership effects. Applying clearer evaluative frameworks and measurable criteria that connect partnership processes to long‐term impacts would enable future reviews to draw more definitive conclusions about how specific principles and strategies translate into meaningful community impacts.

### 4.3 Research Gaps and Future Directions

We identified three key gaps in the literature on rural VHI projects. First, reporting on partnerships was inconsistent, with wide variation in the terminology and detail used to describe processes and effects. While some studies provided comprehensive accounts, others offered minimal information, obscuring shared mechanisms and limiting the ability of partners to learn from one another's partnership experience or from the existing literature. Greater consensus on terminology would enable reviews to more systematically map partnership models, identify common processes and synthesise the current knowledge base. This, in turn, could generate evidence‐based guidance to support partners in making informed decisions about how to approach their partnerships in ways that are tailored to the specific contexts of their ongoing or future work. To address this issue, future research should prioritise more systematic and coherent reporting of partnership engagement and apply established reporting frameworks to support structured and detailed documentation of partnership terminology, processes and effects.

Notably, the included studies describing partnerships with Indigenous communities tended to report unique principles in greater depth and detail, suggesting stronger adherence to existing reporting guidelines or tools (Appendix S7). Partnerships with non‐Indigenous communities could benefit from adopting similar reporting rigour to promote transparency and more accurately reflect their approaches. In addition, advocating for—and engaging in—partnership training and resource development can help researchers and partners build a stronger understanding of partnership standards and best practices in rural and remote VHI projects. Greater familiarity with these approaches would, in turn, enable researchers to more clearly recognise and articulate key processes and effects in their own studies.

The second gap concerned the limited number of studies that formally evaluated their partnerships. Increasing systematic, rigorous evaluations are necessary, as they can clarify the potential benefits and limitations of partnerships, and provide researchers and partners with a stronger rationale for deciding whether, and how, to incorporate partnerships in future projects. Future research should also integrate community perspectives in the evaluation process to ensure outcomes reflect the perspectives and experiences of those most affected and consider longitudinal or follow‐up designs to assess how partnerships develop, adapt and are sustained over time.

Finally, while a variety of principles and strategies are often described, they are usually reported in isolation, without clarifying how principles are translated into concrete, actionable strategies. As a result, it is unclear whether principles function only as abstract values or whether they genuinely guide how partnerships are conducted in practice. Linking specific principles and strategies to reported outcomes would also allow evaluation of how these processes contribute to the success (or lack thereof) of the research partnership project.

### 4.4 Implications

Scientifically, the partnership principles outlined in this review reflect foundational values, norms and expectations that guide researchers and research users toward collaborative practice. These principles shape the ethos of partnerships, informing decision‐making, guiding behaviour and influencing how individuals relate to one another within the research process. The strategies identified offer concrete, action‐oriented approaches that translate these principles into practice, supporting relational dynamics and organisational processes that foster effective engagement and desired outcomes. Together, these principles and strategies represent the *how* of partnerships, offering a flexible set of options that can be adapted to different contexts and study objectives. The outcomes and impacts (the *why* of partnerships) illustrate the value, complexity and challenges of partnership engagement, providing an evidence‐informed rationale for future efforts to integrate partnerships into research. Practically, this review offers guidance on processes and outcomes to support engagement across project planning, development and implementation—key considerations for translating VHI into rural and remote community contexts. This inclusivity fosters early buy‐in, strengthens trust and goodwill, and promotes sustained community engagement and long‐term partnership continuity. Ultimately, by outlining relationship‐centred processes grounded in shared principles and strategies, this review represents a meaningful step toward culturally safe, relevant and sustainable VHI in rural and remote communities, where researchers work *with* and *for* research users.

### 4.5 Strengths and Limitations

First, our findings were constrained by the level of reporting in the included studies. As such, our synthesis reflects only what was documented by the study authors. The analysis was also shaped by the partnership frameworks we applied and the interpretations of our research team. To mitigate these limitations, we engaged in iterative consultations with partners and coauthors (A.S. and C.C.) and sought feedback from external researchers and research users on the clarity, relevance and applicability of our findings. Second, formal reviewer blinding was not used during screening, data extraction, or analysis; therefore, reviewers were not masked to study authors, journals, publication years or affiliations. As a result, reviewer bias cannot be ruled out, although steps were taken to mitigate subjectivity, including independent review, iterative consensus discussions and use of kappa statistics during screening. In addition, the review was limited to peer‐reviewed articles published in English, which may have resulted in the omission of relevant studies published in other languages and may bias findings toward English‐language academic contexts. Finally, grey literature was not included, and the review relied solely on published peer‐reviewed studies.

Third, only a limited number of studies met our inclusion criteria for formal evaluation. Partnerships that did not achieve their anticipated outcomes, such as those facing unexpected challenges, evolving differently than planned, or ending earlier than intended, may also be underrepresented in the literature. We therefore encourage researchers, practitioners and community partners to systematically evaluate their partnership engagement and to transparently report a broad range of outcomes, including challenging or negative ones. Fourth, rurality and remoteness were defined broadly and based on study authors' descriptions. As a result, some relevant studies may have been missed, and we did not assess how differing degrees of rurality or remoteness, such as distance from urban centres, may have influenced partnership processes or outcomes. This remains an important area for future research. Finally, we used AI tools (Copilot) to support the initial drafting of overarching principles, which were then refined and reviewed by team members for accuracy and relevance. While this supported feasibility, we acknowledge limitations given evolving ethical guidelines and the lack of standardised academic practices. Future research could test the use of AI in time‐intensive tasks such as screening and data synthesis, applying standardised protocols and predefined criteria to ensure accuracy and consistency.

This scoping review also had several strengths. First, it was built on a prior scoping review conducted by the principal investigator (F.H.), allowing us to apply lessons, approaches and frameworks from previous studies to improve efficiency and refine our extraction and synthesis methods [6, 19]. Second, we integrated research partnerships as a core component of our process. Throughout the study, from the planning and data collection to dissemination, we engaged in monthly consultations with partners to refine protocols, discuss extraction and analysis, and iteratively shape the final presentation of findings. To identify gaps and future directions, we have incorporated feedback from oral presentations and surveys with relevant research users, ensuring our work remains relevant and aligned with their research and community needs. Third, we emphasised transparency in documenting our research process and findings. Full details of our search strategies, inclusion and exclusion criteria, extraction procedures and analytical steps are provided in the appendices and on the OSF page (https://osf.io/6st9v/).

## 5 Conclusion

This scoping review provided systematic overviews of partnership processes and effects, laying the groundwork for improved guidance for effective partnerships in rural and remote VHI projects. Recognising that meaningful and culturally safe engagement is essential to support both these projects and the communities they serve, we hope this review can ultimately contribute to a reduction in health disparities by improving healthcare access in rural and remote communities through meaningful VHI partnership projects.

## Author Contributions

**Jenna Benbaruj:** formal analysis, validation, investigation, writing – review and editing. **Anurag Singh:** validation, visualization, writing – review and editing. **Carolyn Canfield:** validation, visualization, writing – review and editing. **Emily Thomson:** formal analysis, investigation, validation, writing – original draft, visualization. **Jason Yi:** investigation, writing – original draft, validation, visualization, writing – review and editing, formal analysis, data curation. **Jane Jun:** resources, writing – review and editing. **Femke Hoekstra:** conceptualization, methodology, resources, data curation, funding acquisition, investigation, project administration, formal analysis, validation, writing – review and editing, supervision. **Vania Tjandra:** data curation, formal analysis, investigation, validation, visualization, writing – original draft, writing – review and editing.

## Funding

This work was supported by the University of British Columbia—Hampton Fund Research Grant (F24‐05453; 2024–2026; PI: Hoekstra).

## Disclosure

Guarantor: Femke Hoekstra is the guarantor for this article and accepts full responsibility for the work, had access to the data and controlled the decision to publish.

## Conflicts of Interest

The authors declare no conflicts of interest.

## Supporting information


**Appendix S1:** Partners roles and contribution.
**Appendix S2:** Researchers' positionality.
**Appendix S3:** Abstract/title screening.
**Appendix S4:** Data extraction and analysis: full information.
**Appendix S5:** PRISMA 2020 flow diagram of study selection process.
**Appendix S6:** Reference list of included articles.
**Appendix S7:** Tools and frameworks for guiding partnership engagement and/or reporting of processes and/or effect.
**Appendix S8:** References list 50–139.

## Data Availability

All materials supporting the findings of this study are publicly available via the Open Science Framework (OSF) at https://osf.io/qc4ub/. This includes the full study protocol, inclusion and exclusion criteria, and all files used in data extraction and analysis.
