# Supplementary material for: Research Partnerships in Rural and Remote Virtual Health Innovation Projects: A Scoping Review
Source: Aust J Rural Health. 2026 Jun 8;34(3):e70220. doi: 10.1111/ajr.70220 (PMC13244183; doi:10.1111/ajr.70220)
Supplement: Supplementary file 1 — Appendix S1: Partners roles and contribution. Appendix S2: Researchers' positionality. Appendix S3: Abstract/title screening. Appendix S4: Data extraction and analysis: full information. Appendix S5: PRISMA 2020 flow diagram of study selection process. Appendix S6: Reference list of included articles. Appendix S7: Tools and frameworks for guiding partnership engagement and/or reporting of processes and/or effect. Appendix S8: References list 50–139. [file AJR-34-0-s001.docx]

**Supplementary Appendix 1**

*Partners Roles and Contribution*

Our primary research partners (Carolyn Canfield and Anurag Singh) were engaged from the inception of the project through established collaborations facilitated by senior author Dr. Femke Hoekstra. Both partners, who are also co-authors on this review, were actively involved throughout each stage of the review process. Partner engagement informed aspects of the review process, including refinement of the inclusion criteria, and interpretation and presentation of extracted findings. Engagement occurred through regular email updates, shared documents, and virtual meetings.

Carolyn Canfield, a Citizen-Patient Advisor affiliated with the Innovation Support Unit in the Department of Family Practice, Faculty of Medicine, UBC Vancouver, contributed as a patient partner. Drawing on her experience with co-design and a wide range of collaborative research projects, she provided input on the accessibility and relevance of the findings, the interpretation of results, and the identification of gaps in the literature as well as real-world considerations. Her contributions supported refinements to both the manuscript’s content and presentation, ensuring the review remained responsive to the needs and priorities of patient communities and research users beyond academic settings.

Dr. Anurag Singh, MBBS, PhD, FRCPC, Director of the Northern Centre for Clinical Research and Clinical Assistant Professor in the Department of Medicine, contributed clinical expertise and an applied perspective on hybrid and integrated models of care. His involvement helped ensure that the review’s focus on virtual health innovation reflected real-world healthcare contexts and addressed the priorities of research users working in rural and remote health systems.

To gather broader feedback and diverse perspectives on our preliminary findings, our team presented the findings to the Hybrid Care Research Collaborative (HCRC) during an online session in January 2025. The presentation was attended by a diverse group of researchers and research users interested in the rural health innovation research. Following the session, HCRC members were invited to review the manuscript’s results section and share their feedback and interpretations via a Qualtrics survey (available on OSF). Their insights were used to refine the manuscript’s content, structure, and informed discussion section of the paper, including gaps in the literature and future directions. The names of HCRC members who reviewed the initial version of the manuscript and provided feedback were acknowledged accordingly.

**Supplementary Appendix 2**

***Researchers’ positionality***

**Vania Tjandra (First Author)**

I, Vania Tjandra, the first author, was a third-year undergraduate psychology student at the University of British Columbia Okanagan at the inception of this project. This scoping review represented the first formal research project I undertook in my academic journey after moving to Canada in 2021. The perspective I brought to this review was shaped by both my academic and personal background. As a Chinese-born Indonesian, I grew up appreciating cultural diversity and the importance of collaboration and mutual understanding across different ways of seeing the world. This shaped my appreciation for approaches that move beyond top-down models of support and my belief in the value of research partnerships as a way of working with, rather than for, those most affected, so their voices and lived realities can inform more tailored and meaningful virtual health innovations. In addition, I have lived experience of structural barriers to mental health care, including limited access to appropriate support, as well as experience receiving meaningful support through telehealth. These experiences shaped my attentiveness to barriers that affect access to care, including stigma, limited service pathways, and the importance of support that is responsive to people’s social and cultural contexts. Together, these aspects of my personal background shaped the lens I brought to this review, including a commitment to equitable access and to culturally and contextually responsive virtual health services for underserved communities.

On the academic side, at the time of this project, I was working under the supervision of Dr. Femke Hoekstra within an implementation science and community-oriented research environment. My training in implementation science taught me the importance of research partnerships in a more tangible way, particularly their practical value in supporting implementation and producing meaningful real-world impact. Through hands-on experience working with research team members with diverse expertise and perspectives, including patient partners, researchers, and clinicians, I observed how principles and strategies of research partnership, such as mutual benefit, co-development, trust, and transparent communication, can be enacted in practice. Lastly, my participation in Nowh Guna cultural safety training further shaped my appreciation for listening, relationship-building, trust, and learning from Indigenous histories and perspectives in work involving Indigenous communities.

**Femke Hoekstra (senior author)**

My positionality is shaped by a range of professional and personal experiences related to virtual health research partnerships. I identify as a white, cisgender, straight, married, able‑bodied woman and mother with Dutch nationality. Since 2017, I have lived, worked, and played on the traditional, ancestral, and unceded territory of the Syilx Okanagan Nation.

My interest in research partnership approaches started during my PhD, where I worked in close collaboration with a non‑profit organization and rehabilitation centres to implement a physical activity program in rehabilitative care settings. Through this work, I learned to engage in diverse research partnerships involving individuals and organizations with varied perspectives, roles, and lived experiences. Since then, my research has focused on understanding partnership processes and their impacts across multiple health and community contexts.

I contributed to the co‑development of the Integrated Knowledge Translation (IKT) Guiding Principles and have actively promoted their application in research and practice across different contexts and countries. In my current role as an Assistant Professor in the University of British Columbia’s Department of Medicine, I lead the Implementation Science and Isolated Communities research program. The aim of my research program is to improve healthcare delivery and health services for equity‑deserving populations in rural, remote, and isolated communities. Since the start of my faculty appointment, I have been involved in and have co-led several health innovation initiatives in rural and remote settings, including the Drone Transport Initiative.

While I do not have lived experience of rural or remote communities, I am committed to working in partnership with communities, patients, and other non-academic partners to ensure that the research I conduct is relevant, meaningful, and useful to those who may benefit from it.

***Reflexivity and power dynamics***

This scoping review engaged both researchers and research users with diverse expertise and lived experience, including a patient partner and a rural clinician (see Appendix 1 for details of the partnership). Partners were involved across all stages of the review. From the start to completion, we prioritized transparent and open communication, including check-ins to provide updates and solicit feedback at key points in the process. For example, Zoom meetings were held to share progress and invite partner input on the study protocol and scope, the clarity and appropriateness of the eligibility criteria, and the interpretation of findings. Partner input was incorporated throughout the review, with flexibility in the form and intensity of engagement based on individual availability, interest, and comfort.

Partners made substantive contributions that shaped both the conduct and interpretation of the review. Our patient partner, who brought lived experience and familiarity with the research process, provided important guidance on future research directions and gaps to highlight, including the need to more explicitly incorporate community perspectives into the evaluation of research partnerships. Similarly, a research partner with experience in community-based hybrid care emphasized the importance of recognizing partnership impacts beyond academic outcomes, including community buy-in, trust, and goodwill between researchers and research users.

At the same time, we acknowledge important limitations in representation within our team. Perspectives from rural and remote communities were limited, and although a substantial proportion of included studies focused on partnerships with Indigenous communities, none of our research partners are Indigenous. As a result, our synthesis and interpretation of partnership processes and effects, particularly with respect to Indigenous communities, were informed primarily by the knowledge base, training, and prior experience of non-Indigenous team members. This underscores the need for future research and knowledge synthesis efforts to meaningfully incorporate Indigenous-led and community-led perspectives.

More broadly, our interpretations were shaped by the team’s professional training, academic positioning, and prior experience with virtual health innovation and research partnerships. In addition, as a scoping review, our analysis was necessarily limited to what was reported in the published academic literature, which may not fully capture how partnership processes, decision-making, or outcomes are experienced by partners, particularly community partners, in practice.

Finally, we recognize that, despite commitments to collaborative and inclusive engagement, the extent and nature of partner involvement are often influenced by institutional structures, project timelines, feasibility constraints, and differences in partner capacity or preferences for engagement. These dynamics are frequently underreported in the primary literature and, consequently, may not be fully reflected in this review. Greater transparency in reporting how partners are involved, including the scope, limits, and variability of engagement across project stages, would strengthen both future primary research and knowledge synthesis in this area.

**Supplementary Appendix 3.**

*Abstract/title screening*

During the abstract screening phase, articles were included if they indicated the development, evaluation, and/or implementation of a virtual health innovation and a research partnership approach, defined as at least one health researcher collaborating with any number of research users (e.g., decision or policy makers, healthcare professionals, community organizations, patients, etc.). We chose not to screen for specific populations (i.e., rural, remote, and/or Indigenous) at this stage to avoid prematurely excluding papers that did not mention population details in the abstract.  For article types, we included peer-reviewed, original research articles written in English. Conference or poster abstracts, reviews, editorials, and protocol papers were excluded. Conference or poster abstracts were excluded due to the limited methodological detail and insufficient information available to determine eligibility or extract relevant data. Reviews and secondary synthesis articles were excluded because they often do not provide sufficient detail on specific partnership processes and outcomes of individual virtual health innovation partnerships. This review aimed to examine partnership processes and effects within specific virtual health innovation projects in rural and remote communities. As such, primary studies were required to allow extraction of detailed information on these specific partnership principles, strategies, and outcomes, which are typically not reported in sufficient detail in secondary syntheses. Focusing on primary studies also allowed us to directly examine the original data, rather than relying on interpretations or summaries presented in secondary sources.

Exclusion criteria for abstract screening included the following:

- Lack of indication of both virtual health innovation and a research partnership, as defined (i.e., articles mentioning only virtual health innovation without a partnership approach, or vice versa).
- Not in English.
- Article types such as books, theses, editorials, and conference abstracts.
- Literature reviews or protocols.

 Criteria used to screen title/abstracts can be found in Table 7.

**Table 3.1**

*Inclusion and exclusion criteria for initial screening of title and abstracts*

|  | **Inclusion criteria** | **Exclusion criteria** |
| --- | --- | --- |
| General | - The article describes, reflects and/or evaluates a type of research partnership approach in the area of virtual health research in rural/remote/Indigenous communities. |  |
| Intervention | - The article relates to the development, evaluation and/or implementation of a virtual health innovation that aims to improve healthcare and services in rural/remote/Indigenous communities. - Virtual health innovation is defined as: - *Any technology, method, or approach that leverages digital tools and platforms to improve delivery, accessibility, efficiency, or quality of healthcare services remotely*. *Virtual health innovations include telemedicine platforms, mobile health apps, wearable devices, remote monitoring systems, virtual reality applications for healthcare training and therapy, artificial intelligence-driven diagnostic tools, and online health education resources^1^.* |  |
| Partnership | - The abstract provides an indication that the article meets our definition of health research partnership: - Research partnership is defined as “*individuals, groups or organizations engaged in collaborative research activity involving at least one health researcher (e.g. individual affiliated with an academic institution), and any stakeholder (e.g. decision or policy maker, health care administrator or leader, community agency, charities, network, patients etc.)^2^.*” |  |
| Other | - The article uses quantitative, qualitative, mixed-method research methods or describes a perspective paper. - The article is a commentary paper or case-study. - The article is published in English language. - The article is published in a peer-reviewed journal. | - The article describes a literature review or describes a study protocol. - The article is not published in English language. - Books, theses, editorials, and conference abstracts are excluded. |

**Table 3.2.**

*Detailed inclusion and exclusion criteria for full-text screening*

|  | Inclusion criteria | Exclusion criteria |
| --- | --- | --- |
| Population | - The article focuses on people living in rural/remote communities and/or health professionals/administrators working in rural/remote communities. - Rural/remote communities are defined as *all territory located outside population centers, including urban areas or metropolitan areas^3^*. - The article focuses on Indigenous people and/or health professionals/administrators working in Indigenous communities. - If the article focuses on people living in urban and rural/remote communities, the article must provide specific information on the development, evaluation and/or implementation of health innovation in the rural/remote communities (i.e., the article must clearly indicate that distinct data for rural/remote communities are included and analyzed separately). | - The article focuses on people living in urban centers, or non-rural/remote communities. - The article focuses on people living or working in both urban and rural communities, without a specific focus on targeting people living in rural communities. - The article focuses on marginalized communities, underrepresented groups or other socially isolated communities, without a specific focus on rural/remote communities. |
| Intervention | - The article relates to the development, evaluation and/or implementation of a virtual health innovation that aims to improve healthcare and services in rural/remote/Indigenous communities. - Virtual health innovation is defined as: - *Any technology, method, or approach that leverages digital tools and platforms to improve delivery, accessibility, efficiency, or quality of healthcare services remotely*. *Virtual health innovations include telemedicine platforms, mobile health apps, wearable devices, remote monitoring systems, virtual reality applications for healthcare training and therapy, artificial intelligence-driven diagnostic tools, and online health education resources^1^.* - If a virtual health innovation includes a virtual and non-virtual (e.g., in-person) component, the article must provide specific information on the development, evaluation and/or implementation of the virtual component of the innovation. |  |
| Partnership | - The article meets our definition of health research partnership. - Research partnership is defined as “*individuals, groups or organizations engaged in collaborative research activity involving at least one health researcher (e.g. individual affiliated with an academic institution), and any stakeholder (e.g. decision or policy maker, health care administrator or leader, community agency, charities, network, patients etc.)^2^.*” - The article describes, reflects and/or evaluates a research partnership OR describes, reflects and/or evaluates at least one collaborative research activity. - Collaborative research activity *refers to an activity or moment in the process of planning, conducting, or disseminating research in which there is an indication of shared decision making between at least one researcher and at least one stakeholder^4^.* | - The article does not meet our definition of health research partnership (e.g. physician – patient partnership; student-teacher partnership) or does not provide enough information about the collaborative research activity to determine eligibility. - The article relates to patient engagement in health care decisions instead of research. - Research users are *only* included as participants and not as research partners (e.g. Delphi studies). - If the article describes a research partnership approach or collaborative research activity without reflecting or evaluating it, the article is excluded if: - *all* research users are also *all* participants in the research (100% overlap between participants and research users)* OR - the research users are only engaged in the design of the study and not in the conduct or disseminating phase. |
| Outcomes | - The article describes at least one clear example of a principle that was used or could be used to guide a research partnership team OR at least one clear example of a strategy used to engage research users in the collaborative research activity OR at least one clear example of an outcome/impact of the collaborative research activity. - Principles are defined as “*fundamental norms, rules, or values that represent what is desirable and positive for a person, group, organization, or community, and help it in determining the rightfulness or wrongfulness of its actions. Principles are more basic than policy and objectives and are meant to govern both*”^2^. - Strategies are defined as “*observable actions designed to achieve an outcome*” ^2^. - Outcomes and impacts are defined as *short- or long-term intended and/or unintended changes due directly or indirectly to an intervention. Outcomes are referred to short- or medium-term effects and impacts are referred to long-term or secondary effects^2^.* | - The article does not describe a clear example of a partnership principle or strategy or outcome/impact. |
| Other | - The article uses quantitative, qualitative, mixed-method research methods or describes a perspective paper. - The article is a commentary or case study. - The article is published in English language. - The article is published in a peer-reviewed journal. | - The article describes a literature review or describes a study protocol. - The article is not published in English language. - Books, theses, editorials, and conference abstracts are excluded. |

 Inclusion and exclusion criteria were structured using the PICO framework: Population (P), criteria requiring the involvement of specific research users in virtual health innovation research partnerships; Intervention (I), criteria related to the virtual health innovation itself; Comparison (C), not applicable here; Outcome (O), criteria for reported partnership processes and the effects of research partnerships on virtual health innovation. * We excluded articles describing 100% overlap between research participants and research users when all individuals (e.g., community members) were involved solely as participants, without evidence of collaboration, shared decision-making, or engagement beyond data contribution, including tailoring of the virtual health innovation. This criterion distinguished research partnership approaches from studies in which individuals contributed only as data providers within a primarily researcher-driven process.

**Supplementary Appendix 4.**

*Data extraction and analysis: full information*

Data extraction was guided by the framework of partnership principles, strategies, outcomes, and impacts described previously Hoekstra et al.^6^Data extraction occurred in two phases: general extraction of study and partnership characteristics; and extraction of partnership principles, strategies, and outcomes/impacts.

In the first phase, one team member (FH, ET, JB, JY, or VT) conducted the general extraction of the following partnership characteristics using an Excel spreadsheet (Microsoft, Redmond, WA, USA): first author, year of publication, country of the first author, title, type, and effectiveness of virtual health innovation, aims of the study, and general conclusions. The following partnership characteristics were extracted by four screeners (ET, JB, JY, and VT): partnership key terms, partnership members, definitions or descriptions of the partnership, and level of engagement. Team members consulted the senior author (FH) to resolve any uncertainties encountered during extraction (e.g., partnership definitions, descriptions, study design). The final version was cross-validated between two reviewers (VT and JY). Specifically, the batch of articles initially coded by JY and JB was reviewed by VT, and the batch coded by VT and ET was reviewed by JY. This process ensured independent verification of the extracted data across each reviewer pair.

In the second phase, two team members (ET & VT) extracted partnership principles and strategies using NVivo 14 (Lumivero, Denver, CO, USA) and performed directed qualitative content analysis^38^. Both principles and strategies were extracted only from the methods, results, and/or discussion sections to ensure they were explicitly reflected in the research process. Principles were understood as fundamental beliefs representing what is desirable and positive for a person, group, organization, or community. They help determine the rightness or wrongness of actions within the context of a partnership (e.g., “building and maintaining trusting relationships”). Strategies were defined as observable actions taken within the research process to enact or reflect those principles, or to achieve a particular outcome (e.g., "establishing an advisory group from the local community"). The senior author (FH) independently extracted data from 10 percent of the articles (n = 6) for both partnership principles and strategies, and cross-validated these extractions with the respective team member to ensure consistency. The data classification process used a codebook adapted from a previous review Hoekstra et al., 2023^19^ and a review of reviews Hoekstra et al., 2020^6^. Core classifications were used to categorize principles, strategies, and outcomes/impacts, and subcodes were refined based on the text to ensure that the language and context of the focus of our study reflected the data. Using the same approach, one team member (JY) extracted partnership outcomes/impacts in Excel. For the extraction of outcomes and impacts, only papers that included formal evaluations of the partnership were analyzed. Formal evaluations were defined as those using structured approaches to assess the partnership, such as quantitative rating scales, qualitative interviews, focus groups, or mixed-methods evaluations specifically designed to measure partnership processes or outcomes.

During the initial extraction phase, the identified research partnership processes (strategies and principles) were deductively coded using the exact framing presented in the extracted articles. As the analysis progressed, the subcodes were refined and synthesized to allow a coherent and organized presentation. For outcomes and impacts, minimal changes were made to the codebook adapted from Hoekstra et al. (2020), given the broad nature of these categories. Thus, codes and subcodes for outcomes/impacts were extracted and analyzed based on this existing framework.

All codes and subcodes were transferred to Excel, with each article indicating at least one principle, strategy, and outcome/impact coded as “1” for the corresponding process or effect. Initially, unique subcodes of principles were synthesized into overarching themes using Microsoft Copilot. The senior author (FH) and first author (VT) refined and adapted these overarching partnership principles to ensure they accurately reflected their constituent subcodes, and all team members then reviewed and provided feedback on the findings to finalize the synthesized findings. This process ensured that the synthesized principles accurately reflected the data while remaining accessible and relevant for real-world application. The overarching principles were finalized after review and feedback from other team members.

The findings were presented in a table and divided into two groups: Indigenous or non-Indigenous partnerships (rural and remote communities). Indigenous/Non-Indigenous distinctions were made post hoc based on article content, not as a predefined eligibility criterion to identify potential differences in the reported partnership principles between the two groups, such as the presence of principles uniquely relevant to Indigenous partnerships, recognizing that Indigenous communities may have distinct cultural values, histories, and priorities. The percentage of articles reporting principles and strategies within each classification was calculated to compare reporting frequencies between the two groups. Findings on outcomes and impacts were not categorized by Indigenous or non-Indigenous partnerships due to the small sample size resulting from the limited number of formal evaluation studies.

**Supplementary Appendix 5.**

**Figure 5.1**

*PRISMA 2020 flow diagram of study selection process*


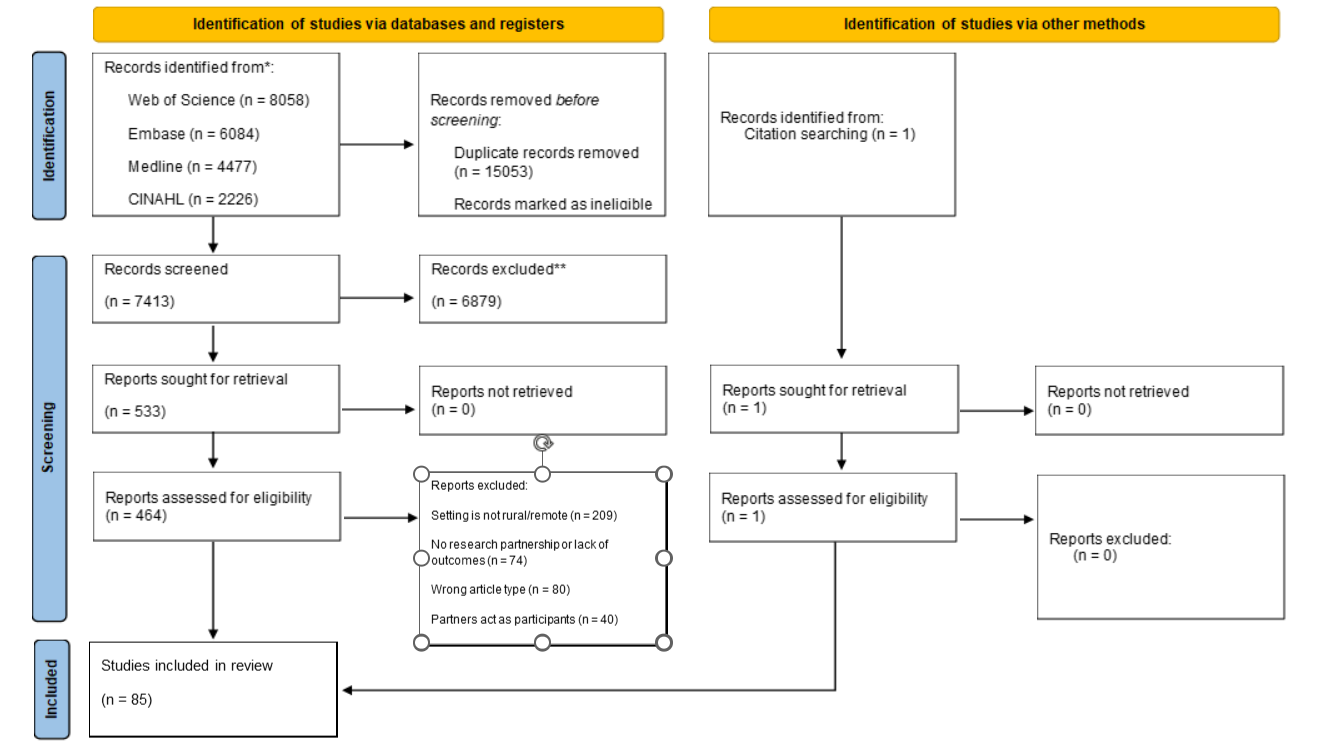
Flow chart adapted from: Page MJ, et al. BMJ 2021;372: n71. doi: 10.1136/bmj.n71.

**Supplementary Appendix 6.**

*Reference list of included articles*

1.  Bishop J, Quilliam C, Wong Shee A. Using integrated knowledge translation to address a rurally based time-critical knowledge gap during the COVID-19 pandemic: a multimethods study in Victoria, Australia. BMJ Open. 2023 Oct;13(10):e075926. Available from: <https://bmjopen.bmj.com/lookup/doi/10.1136/bmjopen-2023-075926>

2. Brown JD, Hales S, Evans TE, Turner T, Sword DO, O’Neil PM, et al. Description, utilisation and results from a telehealth primary care weight management intervention for adults with obesity in South Carolina. J Telemed Telecare. 2020;26(1–2):28–35. Available from: <https://journals.sagepub.com/doi/10.1177/1357633X18789562>

3.       Dahildahil RO, Isip-Tan IT, Marcelo PGF, Grepo L. User-centered Design in Time and Resource-limited Settings: Enhancing the Usability of “Hearing for Life” (HeLe) Device. Acta Med Philipp. 2023;57(9). Available from: <https://doi.org/10.47895/amp.v57i9.1594>

4. Kirkland EB, Dericke D, Bays CC, Wallinger C, McElligott J, Slaughter S, et al. Dissemination of Remote Patient Monitoring: An Academic-Community Primary Care Partnership in South Carolina. J Public Health Manag Pract. 2023;29(4):516–24. Available from: <https://doi.org/10.1097/phh.0000000000001593>

5. Klager E, Lintschinger JM, Teufel A, Schaden E, Manschein V, Reischmann-Senoner L, et al. Optimising co-design processes in telemedicine innovation–developing a telemedical solution for emergency medical services. Shaikh A, editor. PLoS ONE. 2024;19(10):e0309955. Available from: <https://doi.org/10.1371/journal.pone.0309955>

6. Mason JD, Dino G, Boyd J, Crist A, Whanger SL, Edinger K, et al. Amplifying Expertise in Rural West Virginia through Project ECHO: Impactful Partnerships and Community-Engagement. Prog. Community Health Partnersh.: Res. Educ. Action. 2021;15(2):235–42. Available from: <https://doi.org/10.1353/cpr.2021.0025>

7. Miyamoto S, Thiede E, Dorn L, Perkins DF, Bittner C, Scanlon D. The Sexual Assault Forensic Examination Telehealth (SAFE‐T) Center: A Comprehensive, Nurse‐led Telehealth Model to Address Disparities in Sexual Assault Care. J. Rural Health. 2021;37(1):92–102. Available from: <https://onlinelibrary.wiley.com/doi/10.1111/jrh.12474>

8. Ramanadhan S, Ganapathy K, Nukala L, Rajagopalan S, Camillus JC. A model for sustainable, partnership-based telehealth services in rural India: An early process evaluation from Tuver village, Gujarat. Zúniga-González CA, editor. PLoS ONE. 2022;17(1):e0261907. Available from: <https://doi.org/10.1371/journal.pone.0261907>

9. Rahimipour Anaraki N, Mukhopadhyay M, Wilson M, Karaivanov Y, Asghari S. Virtual Healthcare in Rural and Remote Settings: A Qualitative Study of Canadian Rural Family Physicians’ Experiences during the COVID-19 Pandemic. IJERPH;19(20):13397. Available from: <https://doi.org/10.3390/ijerph192013397>

10.       Rohan E, Kuiper N, Bowen S-A, Mast DK, House M, French C, Tharpe FS, Henley SJ, Wanliss E, Puckett M. Pairing Project ECHO and patient navigation as an innovative approach to improving the health and wellness of cancer survivors in rural settings. J Rural Health. 2022; Available from: <https://doi.org/10.1111/jrh.12682>

11. Torres Diaz A, Lock LJ, Molfenter TD, Mahoney JE, Boss D, Bjelland TD, et al. Implementation for Sustained Impact in Teleophthalmology (I-SITE): applying the NIATx Model for tailored implementation of diabetic retinopathy screening in primary care. Implement Sci Commun. 2021;2(1):74. Available from: <https://doi.org/10.1186/s43058-021-00175-0>

12. Tsai C, Damoi JO, Zhang LP, Sarpel U, Binoga M, Kalumuna A, et al. Collaborative international partnership: Enabling the development of a state‐of‐the‐art minimally invasive surgical center in rural Uganda through telementoring. World j surg. 2024;48(7):1602–8. Available from: <https://onlinelibrary.wiley.com/doi/10.1002/wjs.12218>

13. Woodward EN, Oliver KA, Drummond KL, Bartnik MK, McCorkindale A, Meit SS, et al. Transitioning an implementation research intervention to a sustained clinical service: Telehealth primary care mental health integration implementation in Veterans Health Administration. Psychol. Serv. 2024; Available from: <https://doi.apa.org/doi/10.1037/ser0000903>

14. Aronoff-Spencer E, McComsey M, Chih MY, Hubenko A, Baker C, Kim J, et al. Designing a Framework for Remote Cancer Care Through Community Co-design: Participatory Development Study. J Med Internet Res. 2022;24(4):e29492. Available from: <https://doi.org/10.2196/29492>

15. Dabengwa IM, Nyati-Jokomo Z, Chikoko L, Makanga PT, Nyapwere N, Makacha L. A participatory learning approach for the development of a maternal mobile health technology in Zimbabwe. Dev. South. Afr. 2023;40(2):421–40. Available from: <https://www.tandfonline.com/doi/full/10.1080/0376835X.2022.2059449>

16. Fowler LA, Hampl SE, Dreyer Gillette ML, Staiano AE, Kracht CL, Graham AK, et al. Translating Family-Based Behavioral Treatment for Childhood Obesity into a User-Friendly Digital Package for Delivery to Low-Income Families through Primary Care Partnerships: The MO-CORD Study. J. Child. Obes. 2021;17(S1):S-30-S-38. Available from: <https://www.liebertpub.com/doi/10.1089/chi.2021.0174>

17. Guerra AM, De La Vega-Taboada E, Sarmiento OL, Banchoff A, King AC, Stephens D, et al. Fostering collective action for adolescent well-being: citizen science in a Colombian semi-rural area. Health Promot. Int. 2024;39(5):daae144. Available from: <https://academic.oup.com/heapro/article/doi/10.1093/heapro/daae144/7848939>

18.      Kim KK, McGrath SP, Solorza JL, Lindeman D. The ACTIVATE Digital Health Pilot Program for Diabetes and Hypertension in an Underserved and Rural Community. Appl Clin Inform. 2023;14(4):644–53. Available from: <https://doi.org/10.1055/a-2096-0326>

19.      Larson KL, Ballard SM, Ellis DF, Peery JG, Cary JE, Levy RL, Nelson TB, Scroggs LB. A contextual approach to inform a mobile health application for adolescent health. J Child Fam Stud. 2020 Sep;29:3420–32. Available from: <https://doi.org/10.1007/s10826-020-01809-3>

20. Nkangu M, Obegu P, Ngo NV, Wanda F, Sinsai R, Kepgang E, et al. The role of intersectoral collaboration and continuous stakeholder engagement in the implementation of the BornFyne PNMS project in Cameroon. Digit. Health. 2024;10:20552076241287963. Available from: <https://journals.sagepub.com/doi/10.1177/20552076241287963>

21. Obegu P, Nkangu M, Ngo NV, Wanda F, Kasonde M, Kibu OD, et al. Community participation for reproductive, maternal, newborn and child health: insights from the design and implementation of the BornFyne-prenatal management system digital platform in Cameroon. Front Digit Health. 2023;5:1218641. Available from: <https://doi.org/10.3389/fdgth.2023.1218641>

22. O’Donnell EA, Van Citters AD, Khayal IS, Wilson MM, Gustafson D, Barnato AE, et al. A Web-Based Peer Support Network to Help Care Partners of People With Serious Illness: Co-Design Study. JMIR Hum Factors. 2024;11:e53194. Available from: <https://doi.org/10.1177/1049732307313429>

23.     Pozuelo JR, Moffett BD, Davis M, Stein A, Cohen H, Craske MG, et al. User-Centered Design of a Gamified Mental Health App for Adolescents in Sub-Saharan Africa: Multicycle Usability Testing Study. JMIR Form. Res. 2023;7:e51423. Available from: [https://doi.org/10.2196/51423](http://doi.org/10.2196/51423)

24. Sharma P, Tranby B, Kamath C, Brockman TA, Lenhart N, Quade B, et al. Beta test of a Christian faith-based Facebook intervention for smoking cessation in rural communities (FaithCore): development and usability study (preprint). JMIR Form. Res. 2024. Available from: <https://doi.org/10.2196/58121>

25. Veliz Reyes A, Varga MN, Bradwell H, Baxter R, Jones RB, Maudlin D, et al. Codesign principles for the effective development of digital heritage extended reality systems for health interventions in rural communities. CoDesign. 2024;20(2):295–312. Available from: <https://www.tandfonline.com/doi/full/10.1080/15710882.2024.2349588>

26. Wali S, Ssinabulya I, Muhangi CN, Kamarembo J, Atala J, Nabadda M, et al. Bridging community and clinic through digital health: Community-based adaptation of a mobile phone-based heart failure program for remote communities in Uganda. BMC Digit Health. 2023 Jun 16 [cited 2025 Mar 12];1(1):20. Available from: <https://doi.org/10.1186/s44247-023-00020-5>

27. Bugnon B, Geissbuhler A, Bischoff T, Bonnabry P, Von Plessen C. Improving Primary Care Medication Processes by Using Shared Electronic Medication Plans in Switzerland: Lessons Learned From a Participatory Action Research Study. JMIR Form. Res. 2021 Jan 7;5(1):e22319. Available from: <https://doi.org/10.2196/22319>

28. Coury J, Coronado GD, Myers E, Patzel M, Thompson J, Whidden-Rivera C, et al. Engaging with Rural Communities for Colorectal Cancer Screening Outreach Using Modified Boot Camp Translation. Prog. Community Health Partnersh.: Res. Educ. Action. 2024;18(1):47–59. Available from: h[ttps://dx.doi.org/10.1353/cpr.2024.a922329](https://dx.doi.org/10.1353/cpr.2024.a922329).

29. Davis S, Antonio M, Smith M, Burgener P, Lavallee DC, Price M, et al. Paving the Way for Electronic Patient-Centered Measurement in Team-Based Primary Care: Integrated Knowledge Translation Approach. JMIR Form. Res. 2022;6(3):e33584. Available from: <https://doi.org/10.2196/33584>

30. Dindo L, Chaison A, Rodrigues M, Woods K, Mark A, Boykin D. Feasibility of delivering a virtual 1-day acceptance and commitment therapy workshop to rural veterans through community partnerships. Contemp. Clin. Trials Commun. 2023;34:101178. Available from: <https://doi.org/0.1016/j.conctc.2023.101178>

31. Gordon JR, Yack M, Kikuchi K, Stevens L, Merchant L, Buys C, et al. Research-practice partnership: supporting rural cancer survivors in Montana. Cancer Causes Control. 2023;34(12):1085–94. Available from: <https://doi.org/10.1007/s10552-023-01750-7>

32.       Heitkamp TL, Schroeder S, Shogren M, Harsell C. Use of evidence-based technology to assist the behavioral health workforce: a case study on technology transfer systems. J Addict Nurs. 2021 Jul-Sep;32(3):197–204. Available from: https://doi.org/10.1097/JAN.0000000000000418

33. Hinrichs-Kinney LA, Pisegna J, Pontiff ME, Beisheim-Ryan EH, Altic R, Coats H, et al. Mixed-method evaluation to understand clinician perspectives of a program to implement high-intensity resistance rehabilitation into skilled nursing facilities. Arch Phys Med Rehabil. 2025;106(1):61–73. Available from: <https://doi.org/10.1016/j.apmr.2024.09.006>

34. Hoffman MS, Ramsay-Seaner K, Letcher A, Heckmann C. Collaboration is key: implications for successful rural opioid misuse prevention programming. J. Rural Ment Health. 2021;45(3). Available from: <https://doi.org/10.1037/rmh0000184>.

35. Kohn MJ, Chadwick KA, Steinman LE. Adapting Evidence-Based Falls Prevention Programs for Remote Delivery — Implementation Insights through the RE-AIM Evaluation Framework to Promote Health Equity. Prev. Sci. 2024;25(S1):163–73. Available from: <https://doi.org/10.1007/s11121-023-01519-z>

36. Lavender DL, Fleming V, Johnson BR, Southwood R, Prendergast EC, Glenn L, et al. Building Interprofessional Educational Bridges internationally: A reflection on our international partnership to equip future healthcare professionals with skills to care for rural and marginalized populations. Curr. Pharm. Teach. Learn. 2024;16(12):102190. Available from: <https://doi.org/10.1016/j.cptl.2024.102190>

37. Pfeuffer N, Beyer A, Penndorf P, Leiz M, Radicke F, Hoffmann W, et al. Evaluation of a Health Information Exchange System for Geriatric Health Care in Rural Areas: Development and Technical Acceptance Study. JMIR Hum. Factors. 2022;9(3):e34568. Available from: <https://doi.org/10.2196/34568>

38. Please H, Narang K, Bolton W, Nsubuga M, Luweesi H, Richards NB, et al. Virtual reality technology for surgical learning: qualitative outcomes of the first virtual reality training course for emergency and essential surgery delivered by a UK–Uganda partnership. BMJ Open Qual. 2024;13(1):e002477. Available from: <https://qir.bmj.com/lookup/doi/10.1136/bmjoq-2023-002477>

39. Porter KJ, Moon KE, LeBaron VT, Zoellner JM. A Novel Behavioral Intervention for Rural Appalachian Cancer Survivors (weSurvive): Participatory Development and Proof-of-Concept Testing. JMIR Cancer. 2021;7(2):e26010. Available from: <https://doi.org/> [10.2196/26010](https://doi.org/10.2196/26010)

40. Sbaffi L, Zamani E, Kalua K. Promoting Well-being Among Informal Caregivers of People With HIV/AIDS in Rural Malawi: Community-Based Participatory Research Approach. J. Med. Internet Res. 2023;25:e45440. Available from: <https://doi.org/10.2196/45440>

41. Van Pelt S, Massar K, Shields-Zeeman L, De Wit JBF, Van Der Eem L, Lughata AS, et al. The Development of an Electronic Clinical Decision and Support System to Improve the Quality of Antenatal Care in Rural Tanzania: Lessons Learned Using Intervention Mapping. Front Public Health. 2021;9:645521. Available from: <https://doi.org/10.3389/fpubh.2021.645521>

42. Yelton B, Brandt HM, Adams SA, Ureda JR, Lead JR, Fedrick D, et al. “Talk about cancer and build healthy communities”: How visuals are starting the conversation about breast cancer within African-American communities. Int Q Community Health Educ. 2021;41(3):267–74. Available from: <https://doi.org/10.1177/0272684X20942076>

43. Aal T, Ruhl A, Kohler E, Choudhary A, Bhandari P, Devbhankar N, et al. CareConnection – A Digital Caring Community Platform to Overcome Barriers of Asking for, Accepting and Giving Help. In: Mensch und Computer 2023. Rapperswil Switzerland: ACM; 2023. p. 318–24. Available from: <https://dl.acm.org/doi/10.1145/3603555.3608578>

44. Elk R, Emanuel L, Hauser J, Bakitas M, Levkoff S. Developing and testing the feasibility of a culturally based tele-palliative care consult based on the cultural values and preferences of southern, rural African American and White community members: A program by and for the community. Health Equity. 2020;4(1):52–83. Available from: <https://doi.org/10.1089/heq.2019.0120>

45. Holst C, Isabwe GMN, Sukums F, Ngowi H, Kajuna F, Radovanović D, et al. Development of Digital Health Messages for Rural Populations in Tanzania: Multi- and Interdisciplinary Approach. JMIR Mhealth Uhealth. 2021;9(9):e25558. Available from: <https://doi.org/10.2196/25558>

46. Beks, Mitchell, Charles, Wong Shee, Mc Namara, Versace. Implementation of telehealth primary health care services in a rural Aboriginal Community-Controlled Health Organisation during the COVID-19 pandemic: a mixed-methods study. RRH. 2023; Available from: <https://doi.org/10.22605/rrh7521>

47. Dunn KP, Williams KP, Egan CE, Potestio ML, Lee SS. ECHO+: Improving access to hepatitis C care within Indigenous communities in Alberta, Canada. CanLivJ. 2022;5(2):113–23. Available from: <https://canlivj.utpjournals.press/doi/10.3138/canlivj-2021-0027>

48. Leader J, Bighead C, Hunter P, Sanderson R. “Working on a shoestring”: critical resource challenges and place-based considerations for telehealth in Northern Saskatchewan, Canada. J Bioeth Inq. 2023;20:215–23. Available from: <https://doi.org/10.1007/s11673-023-10210-0>

49. Linnaranta O, Cardona LG, Seon Q, Tukkiapik A, Outerbridge J, Bouchard S. Views on a Culturally Safe Psychotherapeutic Treatment by Inuit in Quebec: Co-Design of Cognitive Behavioral Therapy Manual and Virtual Exposure Environments. Cog. Behav. Pract. 2024;S1077722924000671. Available from: <https://doi.org/10.1016/j.cbpra.2024.04.006>

50. Mah J, Pawlovich J, Aldred T, Graham S, Markham R, Williams K, et al. Relational Work Is the Work: Virtual Healthcare Transformation for Rural, Remote and First Nations Communities in British Columbia. Healthc Pap. 2024;21(4):28–37. Available from: <https://doi.org/0.12927/hcpap.2024.27274>

51. McIlduff CD, Acharibasam J, Starr V, Chapados M. Engaging Indigenous older adults with technology use to respond to health and well-being concerns and needs. Healthc. Manage. Forum. 2022 Sep;35(5):257–64. Available from: <https://doi.org/10.1177/08404704221103521>

52. Novak Lauscher H, Blacklaws B, Pritchard E, Wang EJ, Stewart K, Beselt J, et al. Real-Time Virtual Support as an Emergency Department Strategy for Rural, Remote, and Indigenous Communities in British Columbia: Descriptive Case Study. J Med Internet Res. 2023;25:e45451. Available from: <https://doi.org/10.2196/45451>

53. Novak Lauscher H, Stewart K, Markham R, Pawlovich J, Mah J, Hunt M, et al. Real-time virtual supports improving health equity and access in British Columbia. Healthc. Manage. Forum. 2023;36(5):285–92. Available from: <https://journals.sagepub.com/doi/10.1177/08404704231183177>

54. Roach P, Ody M, Campbell P, Bablitz C, Toth E, Murry A, et al. Access, relationships, quality and safety (ARQS): a qualitative study to develop an Indigenous-centred understanding of virtual care quality. BMJ Open Qual. 2022;11(4):e002028. Available from: <https://qir.bmj.com/lookup/doi/10.1136/bmjoq-2022-002028>

55. Roach P, Campbell P, Ody M, Scott M, Barnabe C, Montesanti S, et al. Access, Relationships, Quality and Safety (ARQS): a qualitative study to cocreate an Indigenous patient experience tool for virtual primary care. BMJ Open Qual. 2023;12(4):e002365. Available from: <https://qir.bmj.com/lookup/doi/10.1136/bmjoq-2023-002365>

56. Robler SK, Inglis SM, Gallo JJ, Parnell HE, Ivanoff P, Ryan S, et al. Hearing Norton Sound: community involvement in the design of a mixed methods community randomized trial in 15 Alaska Native communities. Res Involv Engagem. 2020;6(1):67. Available from: <https://doi.org/10.1186/s40900-020-00235-0>

57. Ross JK, Chandorkar AA. Impact of tele-antimicrobial stewardship at two small community hospitals in partnership with an academic medical center: two years of experience. ASHE. 2024;4(1):e145. Available from: [https://doi.org/0.1017/ash.2024.418](https://doi.org/10.1017/ash.2024.418)

58. Young D, Listener L, Ruiz MFT, Chow-Horn W, Lee M, Cutknife L, et al. Ohpikihâwasowin (grounding and guiding on the path to be a healthy parent): virtual adaptation of an Elders mentoring program to support maternal and child wellbeing during the COVID-19 pandemic. BMC Health Serv Res. 2024;24(1):1059. Available from: <https://doi.org/10.1186/s12913-024-11518-7>

59. Batai K, Sanderson PR, Joshweseoma L, Burhansstipanov L, Russell D, Joshweseoma L, et al. Formative Assessment to Improve Cancer Screenings in American Indian Men: Native Patient Navigator and mHealth Texting. IJERPH. 2022;19(11):6546. Available from: <https://doi.org/10.3390/ijerph19116546>

60. Bennett-Levy J, Singer J, Rotumah D, Bernays S, Edwards D. From digital mental health to digital social and emotional wellbeing: how Indigenous community-based participatory research influenced the Australian Government’s digital mental health agenda. Int J Environ Res Public Health. 2021 Sep 16;18(18):9757. Available from: <https://doi.org/10.3390/ijerph18189757>.

61. Britt RK, Britt BC, Anderson J, Fahrenwald N, Harming S. “Sharing Hope and Healing”: A Culturally Tailored Social Media Campaign to Promote Living Kidney Donation and Transplantation Among Native Americans. Health Promot. Pract. 2021;22(6):786–95. Available from: <https://journals.sagepub.com/doi/10.1177/1524839920974580>

62. Glennie M, Dowden M, Grose M, Scolyer M, Superina A, Gardner K. Engaging Remote Aboriginal Communities in COVID-19 Public Health Messaging via Crowdsourcing. Front Public Health. 2022;10:866134. Available from: <https://doi.org/10.3389/fpubh.2022.866134>

63. Healey Akearok G, Tabish T, Cherba M. Cultural orientation and safety app for new and short-term health care providers in Nunavut. Can J Public Health. 2020;111(5):694–700. Available from: <https://doi.org/10.17269/s41997-020-00311-8>

64. Henson C, Chapman F, Shepherd G, Carlson B, Chau JY, Gwynn J, et al. Mature aged Aboriginal and Torres Strait Islander adults are using digital health technologies (original research). Digit. Health. 2022;8:205520762211458. Available from: <http://journals.sagepub.com/doi/10.1177/20552076221145846>

65. Henson C, Chapman F, Shepherd G, Carlson B, Rambaldini B, Gwynne K. Amplifying Older Aboriginal and Torres Strait Islander Women’s Perspectives to Promote Digital Health Equity: Co-Designed Qualitative Study. J Med Internet Res. 2023;25:e50584. Available from: <https://doi.org/10.2196/50584>

66.     Henson C, Freedman B, Rambaldini B, Carlson B, Parter C, Nalliah CJ, Chapman F, Shepherd G, Orchard J, Skinner J, Gwynn J, Macniven R, Ramsden R, Speier SNḴ, Nahdi SM, Christie V, Huang YH, Ward KD, Gwynne K. Wearables are a viable digital health tool for older Indigenous adults living remotely in Australia (research). Digit. Health. 2024;10:20552076241277039. Available from: <https://doi.org/10.1177/20552076241277039>

67. Jacklin K, Pitawanakwat K, Blind M, Lemieux AM, Sobol A, Warry W. Peace of mind: A community-industry-academic partnership to adapt dementia technology for Anishinaabe communities on Manitoulin Island. J. Rehabil. Assist. Technol. Eng. 2020;7:2055668320958327. Available from: <https://journals.sagepub.com/doi/10.1177/2055668320958327>

68. Petrie S, Simard A, Innes E, Kioke S, Groenewoud E, Kozuszko S, et al. Bringing Care Close to Home: Remote Management of Heart Failure In Partnership with Indigenous Communities In Northern Ontario, Canada. CJC Open. 2024;6(12):1423–33. Available from: <https://doi.org/10.1016/j.jcjd.2023.10.085>

69. Roh S, Lee YS, Kenyon DB, Elliott AJ, Petereit DG, Gaba A, et al. Mobile Web App Intervention to Promote Breast Cancer Screening Among American Indian Women in the Northern Plains: Feasibility and Efficacy Study. JMIR Form. Res. 2023;7:e47851. Available from: <https://doi.org/10.2196/47851>

70. Vigil-Hayes M, Collier AF, Hagemann S, Castillo G, Mikkelson K, Dingman J, et al. Integrating Cultural Relevance into a Behavioral mHealth Intervention for Native American Youth. Proc ACM Hum-Comput Interact. 2021;5(CSCW1):1–29. Available from: <https://dl.acm.org/doi/10.1145/3449239>

71.      Binks P, Ross C, Gurruwiwi G, Wurrawilya S, Alley T, Bukulatjpi S, et al. Adapting and translating the ‘Hep B Story’ App the right way: A transferable toolkit to develop health resources with, and for, Aboriginal people. Health Promot J Austr. 2023;35(4):1244–54. Available from: <https://doi.org/10.1002/hpja.858>

72. Blind M, Jacklin K, Pitawanakwat K, Lambrou N, Warry W. Training Indigenous Community Researchers for Community-Based Participatory Ethnographic Dementia Research: A Second-Generation Model. Int. J. Qual. Methods. 2023;22:1–13.Available from: https://[10.1177/16094069231202202](https://doi.org/10.1177/16094069231202202)

73. Cueva K, Cueva M, Revels L, et al. An evaluation of cancer education webinars in Alaska. J. Cancer Educ. 2021;36:484–90. Available from: <https://doi.org/10.1007/s13187-019-01651-x>

74. Devan H, Perry MA, Yaghoubi M, Hale L. “A coalition of the willing”: experiences of co-designing an online pain management programme (iSelf-help) for people with persistent pain. Res Involv Engagem. 2021;7(1):28. Available from: <https://doi.org/10.1186/s40900-021-00275-0>

75. Kerrigan V, Park D, Ross C, Herdman RM, Wilson PM, Gunabarra C, et al. Countering the “wrong story”: a Participatory Action Research approach to developing COVID-19 vaccine information videos with First Nations leaders in Australia. Humanit Soc Sci Commun . 2023;10(1):479. Available from: <https://doi.org/10.1057/s41599-023-01965-8>

76. Nixon P, Broccatelli C, Moss P, Baggio S, Young A, Newcomb D. Healthcare social network research and the ECHO model^TM^: Exploring a community of practice to support cultural brokers and transfer cultural knowledge. BMC Health Serv. Res. 2024;24(1):558. Available from: <https://doi.org/10.1186/s12913-024-11024-w>

77.     Peake RM, Jackson D, Lea J, Usher K. Meaningful engagement with Aboriginal communities using participatory action research to develop culturally appropriate health resources. J Transcult Nurs. 2021 Mar;32(2):129–36. Available from: <https://doi.org/10.1177/1043659619899999>

78. Perry MA, Devan H, Davies C, Hempel D, Ingham T, Jones B, et al. iSelf-Help: a co-designed, culturally appropriate, online pain management programme in Aotearoa. Res Involv Engagem. 2022;8(1):6. Available from: <https://doi.org/10.1186/s40900-022-00339-9>

79. Katapally TR. Smart Indigenous Youth: The Smart Platform Policy Solution for Systems Integration to Address Indigenous Youth Mental Health. JMIR Pediatr Parent. 2020;3(2):e21155. Available from: <https://doi.org/10.2196/21155>

80. Teufel-Shone NI, Goldtooth-Begay C, Begay AB, Lazaro A, Yellowhair J, Todecheenie R, et al. Maintaining the Partnership Between a Tribal Breast and Cervical Cancer Program and a University-Based Cancer Prevention Center During COVID-19 Lock-Down Restrictions-A Case Study. Front Public Health. 2022;10:902253. Available from: <https://doi.org/10.3389/fpubh.2022.902253>

81. Acharibasam JB, Chapados M, Langan J, Starblanket D, Hagel M. Exploring health and wellness with First Nations communities at the “Knowing Your Health Symposium.” Healthc Manage Forum. 2022;35(5):265–71. Available from: <https://doi.org/10.1177/08404704221084042>

82. Povey J, Sweet M, Nagel T, Lowell A, Shand F, Vigona J, et al. Determining Priorities in the Aboriginal and Islander Mental Health Initiative for Youth App Second Phase Participatory Design Project: Qualitative Study and Narrative Literature Review. JMIR Form. Res. 2022;6(2):e28342. Available from: <https://doi.org/10.2196/28342>

83. Rieger KL, Bennett M, Martin D, Hack TF, Cook L, Hornan B. Digital Storytelling as a Patient Engagement and Research Approach With First Nations Women: How the Medicine Wheel Guided Our Debwewin Journey. Qual. Health Res. 2021;31(12):2163–75. Available from: <https://doi.org/10.1177/10497323211027529>

84. Sauvé A, Cappelletti A, Murji L. Stand Up for Indigenous Health: A Simulation to Educate Residents About the Social Determinants of Health Faced by Indigenous Peoples in Canada. Acad Med. 2022;97(4):518–23. Available from: <https://doi.org/10.1097/acm.0000000000004570>

85. Snijder M, Stapinski L, Ward J, Lees B, Chapman C, Champion K, et al. Strong and Deadly Futures: Co-Development of a Web-Based Wellbeing and Substance Use Prevention Program for Aboriginal and Torres Strait Islander and Non-Aboriginal Adolescents. IJERPH. 2021;18(4):2176. Available from: <https://doi.org/10.3390/ijerph1804217>

**Supplementary Appendix 7.**

**Table 7.1**

*Tools and Frameworks for Guiding Partnership Engagement and/or Reporting of Processes and/or Effect*

| **TMFA** | **Classification** | | **Purpose/Description** | | **List of Studies (First Author)** |
| --- | --- | --- | --- | --- | --- |
| **Ownership, Control, Access, and Possession (OCAP)** | | Framework | A set of principles—Ownership, Control, Access, and Possession (OCAP)—that assert First Nations' right to govern research involving their communities. Researchers can use the OCAP framework to guide engagement with Indigenous communities, as it offers concrete strategies such as upskilling community members, community-led review processes, consideration of community capacity and commitment, and data governance protocols. These strategies aim to support ethical, reciprocal, and culturally safe research, with potential benefits including capacity-building, community relevance, and self-determination. Researchers can also use OCAP to guide the reporting of partnership processes and outcomes by assessing how their research has incorporated these principles and strategies, achieved related impacts, and documented community engagement accordingly^5^. | | Roach et. al.^90^  Roach et. al.^91^ |
| **Consolidated Criteria for Strengthening Reporting of Health Research (CONSIDER)** | | Framework | A reporting guideline developed through a collaborative, Indigenous-led process to strengthen the quality and transparency of health research involving Indigenous Peoples. The CONSIDER framework outlines eight research domains and 17 criteria—including governance, relationships, prioritization, and capacity—and provides a checklist that researchers can use to plan, conduct, and report on research partnerships with Indigenous communities. It offers a practical tool for documenting ethical engagement and strengthening partnership outcomes in health research contexts^8^. | | Beks, et. al.^82^ |
| **The Guidance for Reporting Involvement of Patients and the Public (GRIPP2)** | | Framework | The Guidance for Reporting Involvement of Patients and the Public (GRIPP2) includes two evidence-based checklists—long form (LF) and short form (SF)—developed through international consensus to improve the quality, transparency, and consistency of reporting on patient and public involvement (PPI) in research. GRIPP2-LF is intended for studies that centrally incorporate a PPI approach, while the SF is used when PPI is a secondary focus. Both tools help researchers document how involvement was conducted, its context, impact, and outcomes, offering a structured approach to reporting partnership processes and effects^10^. | Perry et. al.^112^ | |
| **The SMART Framework** | | Framework | An evidence-based framework that integrates citizen science, community-based participatory research, and systems science through smartphone-enabled tools. The SMART Framework provides a structured approach to equitable partnerships by emphasizing continuous stakeholder engagement, shared governance, and co-creation of data and knowledge throughout the research process. While not a formal reporting checklist, using the framework can help researchers evaluate whether and how these collaborative processes were incorporated, supporting more systematic documentation and reflection on engagement^11^. | Katapally^113^ | |
| **The Partnership Pentagram Plus (PPP)** | | Framework | The Partnership Pentagram Plus (PPP) framework provides a structured, relationship-centred approach to socially accountable health system change. Developed in collaboration with Indigenous partners in British Columbia, it guides researchers in engaging diverse perspectives through processes such as appreciative inquiry, deliberative dialogue, and two-eyed seeing. With its emphasis on simultaneity, shared governance, and mutual accountability, the framework can support structured reflection and documentation—helping researchers evaluate whether and how inclusive co-creation was achieved, and identify changes resulting from partnership engagement, such as stronger, trusting relationships, more culturally grounded care, and better access to services^13^. | | Mah et. al.^86^ |
| **Appreciative Inquiry (AI)** | | Model | Researchers can use the Appreciative Inquiry (AI) model to structure partnership engagement through four phases: Discover, Dream, Design, and Deliver. This approach helps identify partner strengths and needs, fosters collaborative goal setting, and guides actionable planning with a focus on feasibility and translation of innovation. By following this structured process, researchers can better document and evaluate their engagement, tracking the process of engagement and progress throughout each distinct phase, such as identifying which partnership processes were incorporated in each phase and their resulting effects^15^. | | Snijder et. al.^119^ |
| **RE-AIM** | | Framework | A planning and evaluation framework—RE-AIM (Reach, Effectiveness, Adoption, Implementation, and Maintenance)—that supports researchers in assessing the real-world impact of health interventions across multiple levels. Researchers can use the RE-AIM framework to guide partnership-engaged implementation research, as it offers structured dimensions to evaluate outcomes such as who the intervention reached, how effectively it worked, whether it was adopted by partners, how it was implemented (including adaptations), and whether it was sustained over time. RE-AIM can be used to evaluate how partnerships influence implementation outcomes—supporting more systematic and outcomes-focused assessment of collaborative engagement^17^. | | Kohn et. al.^71^ |
| **The Rambaldini Model of Collective Impact** | | Model | The Rambaldini Model of Collective Impact provides a structured approach to co-design through clearly defined preconditions and stages, including shared measurement, continuous communication, and power-sharing. As a result, researchers adopting this framework may be better supported in documenting co-design processes with partners—for example, by clarifying what partnership activities are incorporated, how they are carried out at each research stage, and how decisions are made. This structured engagement encourages more consistent and transparent documentation of partnership processes and outcomes in Aboriginal health research^19^. | | Henson et. al.^101^    Henson et. al.^102^ |

**Supplementary Appendix 8.**

*References list 50-138*

52. Woodward EN, Oliver KA, Drummond KL, Bartnik MK, McCorkindale A, Meit SS, et al. Transitioning an implementation research intervention to a sustained clinical service: Telehealth primary care mental health integration implementation in Veterans Health Administration. Psychol Serv. 2024. Available from:<https://doi.apa.org/doi/10.1037/ser0000903>

53. Aronoff-Spencer E, McComsey M, Chih MY, Hubenko A, Baker C, Kim J, et al. Designing a framework for remote cancer care through community co-design: Participatory development study. J Med Internet Res. 2022;24(4):e29492. Available from:<https://doi.org/10.2196/29492>

54. Fowler LA, Hampl SE, Dreyer Gillette ML, Staiano AE, Kracht CL, Graham AK, et al. Translating family-based behavioral treatment for childhood obesity into a user-friendly digital package for delivery to low-income families through primary care partnerships: The MO-CORD study. Child Obes. 2021;17(S1):S30–8. Available from:<https://doi.org/10.1089/chi.2021.0174>

55. Guerra AM, De La Vega-Taboada E, Sarmiento OL, Banchoff A, King AC, Stephens D, et al. Fostering collective action for adolescent well-being: Citizen science in a Colombian semi-rural area. Health Promot Int. 2024;39(5):daae144. Available from:<https://doi.org/10.1093/heapro/daae144>

56. Kim KK, McGrath SP, Solorza JL, Lindeman D. The ACTIVATE digital health pilot program for diabetes and hypertension in an underserved and rural community. Appl Clin Inform. 2023;14(4):644–53. Available from:<https://doi.org/10.1055/a-2096-0326>

57. Larson KL, Ballard SM, Ellis DF, Peery JG, Cary JE, Levy RL, et al. A contextual approach to inform a mobile health application for adolescent health. J Child Fam Stud. 2020 Sep;29:3420–32. Available from:<https://doi.org/10.1007/s10826-020-01809-3>

58. Nkangu M, Obegu P, Ngo NV, Wanda F, Sinsai R, Kepgang E, et al. The role of intersectoral collaboration and continuous stakeholder engagement in the implementation of the BornFyne PNMS project in Cameroon. Digit Health. 2024;10:20552076241287963. Available from:<https://doi.org/10.1177/20552076241287963>

59. Obegu P, Nkangu M, Ngo NV, Wanda F, Kasonde M, Kibu OD, et al. Community participation for reproductive, maternal, newborn and child health: Insights from the design and implementation of the BornFyne-prenatal management system digital platform in Cameroon. Front Digit Health. 2023;5:1218641. Available from:<https://doi.org/10.3389/fdgth.2023.1218641>

60. O’Donnell EA, Van Citters AD, Khayal IS, Wilson MM, Gustafson D, Barnato AE, et al. A web-based peer support network to help care partners of people with serious illness: Co-design study. JMIR Hum Factors. 2024;11:e53194. Available from:<https://doi.org/10.2196/53194>

61. Sharma P, Tranby B, Kamath C, Brockman TA, Lenhart N, Quade B, et al. Beta test of a Christian faith-based Facebook intervention for smoking cessation in rural communities (FaithCore): Development and usability study (preprint). JMIR Form Res. 2024. Available from:<https://doi.org/10.2196/58121>

62. Veliz Reyes A, Varga MN, Bradwell H, Baxter R, Jones RB, Maudlin D, et al. Codesign principles for the effective development of digital heritage extended reality systems for health interventions in rural communities. CoDesign. 2024;20(2):295–312. Available from:<https://www.tandfonline.com/doi/full/10.1080/15710882.2024.2349588>

63. Wali S, Ssinabulya I, Muhangi CN, Kamarembo J, Atala J, Nabadda M, et al. Bridging community and clinic through digital health: Community-based adaptation of a mobile phone-based heart failure program for remote communities in Uganda. BMC Digit Health. 2023;1(1):20. Available from:<https://doi.org/10.1186/s44247-023-00020-5>

64. Bugnon B, Geissbuhler A, Bischoff T, Bonnabry P, Von Plessen C. Improving primary care medication processes by using shared electronic medication plans in Switzerland: Lessons learned from a participatory action research study. JMIR Form Res . 2021;5(1):e22319. Available from:<https://doi.org/10.2196/22319>

65. Coury J, Coronado GD, Myers E, Patzel M, Thompson J, Whidden-Rivera C, et al. Engaging with rural communities for colorectal cancer screening outreach using modified boot camp translation. Prog Community Health Partnersh. 2024;18(1):47–59. Available from:<https://doi.org/10.1353/cpr.2024.a922329>

66. Davis S, Antonio M, Smith M, Burgener P, Lavallee DC, Price M, et al. Paving the way for electronic patient-centered measurement in team-based primary care: Integrated knowledge translation approach. JMIR Form Res. 2022;6(3):e33584. Available from:<https://doi.org/10.2196/33584>

67. Dindo L, Chaison A, Rodrigues M, Woods K, Mark A, Boykin D. Feasibility of delivering a virtual 1-day acceptance and commitment therapy workshop to rural veterans through community partnerships. Contemp Clin Trials Commun. 2023;34:101178. Available from:<https://doi.org/10.1016/j.conctc.2023.101178>

68. Gordon JR, Yack M, Kikuchi K, Stevens L, Merchant L, Buys C, et al. Research-practice partnership: Supporting rural cancer survivors in Montana. Cancer Causes Control. 2023;34(12):1085–94. Available from:<https://doi.org/10.1007/s10552-023-01750-7>

69. Heitkamp T, Schroeder S, Shogren M, Harsell C. Use of evidence-based technology to assist the behavioral health workforce: A case study on technology transfer systems. J Addict Nurs. 2021 Jul–Sep;32(3):197–204. Available from:<https://doi.org/10.1097/JAN.0000000000000418>

70. Hinrichs-Kinney LA, Pisegna J, Pontiff ME, Beisheim-Ryan EH, Altic R, Coats H, et al. Mixed-method evaluation to understand clinician perspectives of a program to implement high-intensity resistance rehabilitation into skilled nursing facilities. Arch Phys Med Rehabil. 2025;106(1):61–73. Available from:<https://doi.org/10.1016/j.apmr.2024.09.006>

71. Hoffman MS, Ramsay-Seaner K, Letcher A, Heckmann C. Collaboration is key: Implications for successful rural opioid misuse prevention programming. J Rural Ment Health. 2021 May;45(3):198–206. Available from:<https://doi.org/10.1037/rmh0000184>

72. Kohn MJ, Chadwick KA, Steinman LE. Adapting evidence-based falls prevention programs for remote delivery: Implementation insights through the RE-AIM evaluation framework to promote health equity. Prev Sci. 2024;25(S1):163–73. Available from:<https://doi.org/10.1007/s11121-023-01519-z>

73. Lavender DL, Fleming V, Johnson BR, Southwood R, Prendergast EC, Glenn L, et al. Building interprofessional educational bridges internationally: A reflection on our international partnership to equip future healthcare professionals with skills to care for rural and marginalized populations. Curr Pharm Teach Learn. 2024;16(12):102190. Available from:<https://doi.org/10.1016/j.cptl.2024.102190>

74. Pfeuffer N, Beyer A, Penndorf P, Leiz M, Radicke F, Hoffmann W, et al. Evaluation of a health information exchange system for geriatric health care in rural areas: Development and technical acceptance study. JMIR Hum Factors. 2022 Sep 15;9(3):e34568. Available from:<https://doi.org/10.2196/34568>

75. Please H, Narang K, Bolton W, Nsubuga M, Luweesi H, Richards NB, et al. Virtual reality technology for surgical learning: Qualitative outcomes of the first virtual reality training course for emergency and essential surgery delivered by a UK–Uganda partnership. BMJ Open Qual. 2024;13(1):e002477. Available from:<https://doi.org/10.1136/bmjoq-2023-002477>

76. Porter KJ, Moon KE, LeBaron VT, Zoellner JM. A novel behavioral intervention for rural Appalachian cancer survivors (weSurvive): Participatory development and proof-of-concept testing. JMIR Cancer. 2021;7(2):e26010. Available from:<https://doi.org/10.2196/26010>

77. Sbaffi L, Zamani E, Kalua K. Promoting well-being among informal caregivers of people with HIV/AIDS in rural Malawi: Community-based participatory research approach. J Med Internet Res. 2023;25:e45440. Available from:<https://doi.org/10.2196/45440>

78. Van Pelt S, Massar K, Shields-Zeeman L, De Wit JBF, Van Der Eem L, Lughata AS, et al. The development of an electronic clinical decision and support system to improve the quality of antenatal care in rural Tanzania: Lessons learned using intervention mapping. Front Public Health. 2021;9:645521. Available from:<https://doi.org/10.3389/fpubh.2021.645521>

79. Yelton B, Brandt HM, Adams SA, Ureda JR, Lead JR, Fedrick D, et al. “Talk about cancer and build healthy communities”: How visuals are starting the conversation about breast cancer within African-American communities. Int Q Community Health Educ. 2021;41(3):267–74. Available from:<https://doi.org/10.1177/0272684X20942076>

80. Aal T, Ruhl A, Kohler E, Choudhary A, Bhandari P, Devbhankar N, et al. CareConnection – A digital caring community platform to overcome barriers of asking for, accepting and giving help. In: Mensch und Computer 2023. Rapperswil, Switzerland: ACM; 2023. p. 318–24. Available from:<https://doi.org/10.1145/3603555.3608578>

81. Elk R, Emanuel L, Hauser J, Bakitas M, Levkoff S. Developing and testing the feasibility of a culturally based tele-palliative care consult based on the cultural values and preferences of southern, rural African American and White community members: A program by and for the community. Health Equity. 2020;4(1):52–83. Available from:<https://doi.org/10.1089/heq.2019.0120>

82. Holst C, Isabwe GMN, Sukums F, Ngowi H, Kajuna F, Radovanović D, et al. Development of digital health messages for rural populations in Tanzania: Multi- and interdisciplinary approach. JMIR Mhealth Uhealth. 2021;9(9):e25558. Available from:<https://doi.org/10.2196/25558>

83. Beks H, Mitchell C, Charles S, Wong Shee A, Mc Namara KP, Versace VL. Implementation of telehealth primary health care services in a rural Aboriginal Community-Controlled Health Organisation during the COVID-19 pandemic: A mixed-methods study. Rural Remote Health. 2023. Available from:<https://doi.org/10.22605/RRH7521>

84. Dunn KP, Williams KP, Egan CE, Potestio ML, Lee SS. ECHO+: Improving access to hepatitis C care within Indigenous communities in Alberta, Canada. Can Liver J. 2022;5(2):113–23. Available from:<https://canlivj.utpjournals.press/doi/10.3138/canlivj-2021-0027>

85. Leader J, Bighead C, Hunter P, Sanderson R. “Working on a shoestring”: Critical resource challenges and place-based considerations for telehealth in Northern Saskatchewan, Canada. J Bioeth Inq. 2023 Feb;20:215–23. Available from:<https://doi.org/10.1007/s11673-023-10210-0>

86. Linnaranta O, Cardona LG, Seon Q, Tukkiapik A, Outerbridge J, Bouchard S. Views on a culturally safe psychotherapeutic treatment by Inuit in Quebec: Co-design of cognitive behavioral therapy manual and virtual exposure environments. Cogn Behav Pract. 2024;S1077722924000671. Available from:<https://doi.org/10.1016/j.cbpra.2024.04.006>

87. Mah J, Pawlovich J, Aldred T, Graham S, Markham R, Williams K, et al. Relational work is the work: Virtual healthcare transformation for rural, remote and First Nations communities in British Columbia. Healthc Policy. 2024;21(4):28–37. Available from:<https://doi.org/10.12927/hcpap.2024.27274>

88. McIlduff CD, Acharibasam J, Starr V, Chapados M. Engaging Indigenous older adults with technology use to respond to health and well-being concerns and needs. Healthc Manage Forum. 2022;35(5):257–64. Available from:<https://doi.org/10.1177/08404704221103521>

89. Novak Lauscher H, Blacklaws B, Pritchard E, Wang EJ, Stewart K, Beselt J, et al. Real-time virtual support as an emergency department strategy for rural, remote, and Indigenous communities in British Columbia: Descriptive case study. J Med Internet Res. 2023;25:e45451. Available from:<https://doi.org/10.2196/45451>

90. Novak Lauscher H, Stewart K, Markham R, Pawlovich J, Mah J, Hunt M, et al. Real-time virtual supports improving health equity and access in British Columbia. Healthc Manage Forum. 2023;36(5):285–92. Available from:<https://doi.org/10.1177/08404704231183177>

91. Roach P, Ody M, Campbell P, Bablitz C, Toth E, Murry A, et al. Access, relationships, quality and safety (ARQS): A qualitative study to develop an Indigenous-centred understanding of virtual care quality. BMJ Open Qual. 2022;11(4):e002028. Available from:<https://doi.org/10.1136/bmjoq-2022-002028>

92. Roach P, Campbell P, Ody M, Scott M, Barnabe C, Montesanti S, et al. Access, relationships, quality and safety (ARQS): A qualitative study to cocreate an Indigenous patient experience tool for virtual primary care. BMJ Open Qual. 2023;12(4):e002365. Available from:<https://doi.org/10.1136/bmjoq-2023-002365>

93. Robler SK, Inglis SM, Gallo JJ, Parnell HE, Ivanoff P, Ryan S, et al. Hearing Norton Sound: Community involvement in the design of a mixed methods community randomized trial in 15 Alaska Native communities. Res Involv Engagem. 2020;6(1):67. Available from:<https://doi.org/10.1186/s40900-020-00235-0>

94. Ross JK, Chandorkar AA. Impact of tele-antimicrobial stewardship at two small community hospitals in partnership with an academic medical center: Two years of experience. Antimicrob Steward Healthc Epidemiol. 2024;4(1):e145. Available from:<https://doi.org/10.1017/ash.2024.418>

95. Young D, Listener L, Ruiz MFT, Chow-Horn W, Lee M, Cutknife L, et al. Ohpikihâwasowin (grounding and guiding on the path to be a healthy parent): Virtual adaptation of an Elders mentoring program to support maternal and child wellbeing during the COVID-19 pandemic. BMC Health Serv Res. 2024;24(1):1059. Available from:<https://doi.org/10.1186/s12913-024-11518-7>

96. Batai K, Sanderson PR, Joshweseoma L, Burhansstipanov L, Russell D, Joshweseoma L, et al. Formative assessment to improve cancer screenings in American Indian men: Native patient navigator and mHealth texting. Int J Environ Res Public Health . 2022;19(11):6546. Available from:<https://doi.org/10.3390/ijerph19116546>

97. Bennett-Levy J, Singer J, Rotumah D, Bernays S, Edwards D. From digital mental health to digital social and emotional wellbeing: How Indigenous community-based participatory research influenced the Australian Government’s digital mental health agenda. Int J Environ Res Public Health. 2021 Sep 16;18(18):9757. Available from:<https://doi.org/10.3390/ijerph18189757>

98. Britt RK, Britt BC, Anderson J, Fahrenwald N, Harming S. “Sharing hope and healing”: A culturally tailored social media campaign to promote living kidney donation and transplantation among Native Americans. Health Promot Pract. 2021;22(6):786–95. Available from:<https://doi.org/10.1177/1524839920974580>

99. Glennie M, Dowden M, Grose M, Scolyer M, Superina A, Gardner K. Engaging remote Aboriginal communities in COVID-19 public health messaging via crowdsourcing. Front Public Health. 2022;10:866134. Available from:<https://doi.org/10.3389/fpubh.2022.866134>

100. Akearok G, Tabish T, Cherba M. Cultural orientation and safety app for new and short-term health care providers in Nunavut. Can J Public Health . 2020;111(5):694–700. Available from: https://doi.org/10.17269/s41997-020-00311-8

101. Henson C, Chapman F, Shepherd G, Carlson B, Chau JY, Gwynn J, et al. Mature aged Aboriginal and Torres Strait Islander adults are using digital health technologies (original research). Digit Health. 2022;8:205520762211458. Available from:<https://doi.org/10.1177/20552076221145846>

102. Henson C, Chapman F, Shepherd G, Carlson B, Rambaldini B, Gwynne K. Amplifying older Aboriginal and Torres Strait Islander women’s perspectives to promote digital health equity: Co-designed qualitative study. J Med Internet Res. 2023;25:e50584. Available from:<https://doi.org/10.2196/50584>

103. Henson C, Freedman B, Rambaldini B, Carlson B, Parter C, Nalliah CJ, et al. Wearables are a viable digital health tool for older Indigenous adults living remotely in Australia (research). Digit Health. 2024;10:20552076241277039. Available from:<https://doi.org/10.1177/20552076241277039>

104. Jacklin K, Pitawanakwat K, Blind M, Lemieux AM, Sobol A, Warry W. Peace of mind: A community–industry–academic partnership to adapt dementia technology for Anishinaabe communities on Manitoulin Island. J Rehabil Assist Technol Eng. 2020;7:2055668320958327. Available from:<https://doi.org/10.1177/2055668320958327>

105. Petrie S, Simard A, Innes E, Kioke S, Groenewoud E, Kozuszko S, et al. Bringing care close to home: Remote management of heart failure in partnership with Indigenous communities in Northern Ontario, Canada. CJC Open. 2024;6(12):1423–33. Available from:<https://doi.org/10.1016/j.cjco.2023.10.085>

106. Roh S, Lee YS, Kenyon DB, Elliott AJ, Petereit DG, Gaba A, et al. Mobile web app intervention to promote breast cancer screening among American Indian women in the Northern Plains: Feasibility and efficacy study. JMIR Form Res . 2023;7:e47851. Available from:<https://doi.org/10.2196/47851>

107. Vigil-Hayes M, Collier AF, Hagemann S, Castillo G, Mikkelson K, Dingman J, et al. Integrating cultural relevance into a behavioral mHealth intervention for Native American youth. Proc ACM Hum Comput Interact. 2021;5(CSCW1):1–29. Available from:<https://doi.org/10.1145/3449239>

108. Cueva K, Cueva M, Revels L, et al. An evaluation of cancer education webinars in Alaska. J Cancer Educ. 2021 Jun;36:484–90. Available from:<https://doi.org/10.1007/s13187-019-01651-x>

109. Devan H, Perry MA, Yaghoubi M, Hale L. “A coalition of the willing”: Experiences of co-designing an online pain management programme (iSelf-help) for people with persistent pain. Res Involv Engagem. 2021;7(1):28. Available from:<https://doi.org/10.1186/s40900-021-00275-0>

110. Kerrigan V, Park D, Ross C, Herdman RM, Wilson PM, Gunabarra C, et al. Countering the “wrong story”: A participatory action research approach to developing COVID-19 vaccine information videos with First Nations leaders in Australia. Humanit Soc Sci Commun. 2023 Aug;10(1):479. Available from:<https://doi.org/10.1057/s41599-023-01965-8>

111. Nixon P, Broccatelli C, Moss P, Baggio S, Young A, Newcomb D. Healthcare social network research and the ECHO model™: Exploring a community of practice to support cultural brokers and transfer cultural knowledge. BMC Health Serv Res. 2024;24(1):558. Available from:<https://doi.org/10.1186/s12913-024-11024-w>

112. Peake RM, Jackson D, Lea J, Usher K. Meaningful engagement with Aboriginal communities using participatory action research to develop culturally appropriate health resources. J Transcult Nurs. 2021 Mar;32(2):129–36. Available from:<https://doi.org/10.1177/1043659619899999>

113. Perry MA, Devan H, Davies C, Hempel D, Ingham T, Jones B, et al. iSelf-help: A co-designed, culturally appropriate, online pain management programme in Aotearoa. Res Involv Engagem. 2022;8(1):6. Available from:<https://doi.org/10.1186/s40900-022-00339-9>

114. Katapally TR. Smart Indigenous Youth: The Smart Platform policy solution for systems integration to address Indigenous youth mental health. JMIR Pediatr Parent. 2020;3(2):e21155. Available from:<https://doi.org/10.2196/21155>

115. Teufel-Shone NI, Goldtooth-Begay C, Begay AB, Lazaro A, Yellowhair J, Todecheenie R, et al. Maintaining the partnership between a tribal breast and cervical cancer program and a university-based cancer prevention center during COVID-19 lock-down restrictions: A case study. Front Public Health. 2022;10:902253. Available from:<https://doi.org/10.3389/fpubh.2022.902253>

116. Acharibasam JB, Chapados M, Langan J, Starblanket D, Hagel M. Exploring health and wellness with First Nations communities at the “Knowing Your Health Symposium.” Healthc Manage Forum. 2022;35(5):265–71. Available from:<https://doi.org/10.1177/08404704221084042>

117. Povey J, Sweet M, Nagel T, Lowell A, Shand F, Vigona J, et al. Determining priorities in the Aboriginal and Islander Mental Health Initiative for Youth App second phase participatory design project: Qualitative study and narrative literature review. JMIR Form Res. 2022;6(2):e28342. Available from:<https://doi.org/10.2196/28342>

118. Rieger KL, Bennett M, Martin D, Hack TF, Cook L, Hornan B. Digital storytelling as a patient engagement and research approach with First Nations women: How the medicine wheel guided our Debwewin journey. Qual Health Res. 2021;31(12):2163–75. Available from:<https://doi.org/10.1177/10497323211027529>

119. Sauvé A, Cappelletti A, Murji L. Stand up for Indigenous health: A simulation to educate residents about the social determinants of health faced by Indigenous peoples in Canada. Acad Med. 2022;97(4):518–23. Available from:<https://doi.org/10.1097/ACM.0000000000004570>

120. Snijder M, Stapinski L, Ward J, Lees B, Chapman C, Champion K, et al. Strong and Deadly Futures: Co-development of a web-based wellbeing and substance use prevention program for Aboriginal and Torres Strait Islander and non-Aboriginal adolescents. Int J Environ Res Public Health. 2021;18(4):2176. Available from:<https://doi.org/10.3390/ijerph1804217>

121. Brant H, Atherton H, Ziebland S, McKinstry B, Campbell JL, Salisbury C. Using alternatives to face-to-face consultations: A survey of prevalence and attitudes in general practice. Br J Gen Pract. 2016;66(648):e460–6. Available from:<https://doi.org/10.3399/bjgp16X685597>

122. Golinelli D, Boetto E, Carullo G, Nuzzolese AG, Landini MP, Fantini MP. Adoption of digital technologies in health care during the COVID-19 pandemic: Systematic review of early scientific literature. J Med Internet Res. 2020;22(11):e22280. Available from:<https://doi.org/10.2196/22280>

123. Jones L, Jacklin K, O’Connell ME. Development and use of health-related technologies in Indigenous communities: Critical review. J Med Internet Res. 2017;19(7):e256. Available from:<https://doi.org/10.2196/jmir.7520>

124. Minniecon D, Franks N, Heffernan M. Indigenous research: Three researchers reflect on their experiences at the interface. Australas Psychiatry. 2007;36(S1). Available from:<https://doi.org/10.1017/S132601110000466X>

125. Australian Institute of Aboriginal and Torres Strait Islander Studies. Guidelines for ethical research in Indigenous studies. Aust Indig Law Rep. 2003;8(1):85–94. Available from:<https://www.jstor.org/stable/26479543>

126. Hart MA. Indigenous worldviews, knowledge, and research: The development of an Indigenous research paradigm. J Indig Voices Soc Work. 2010;1(1A). Available from:<https://journalhosting.ucalgary.ca/index.php/jivsws/article/view/63043>

127. Pelletier CA, Pousette A, Ward K, Fox G. Exploring the perspectives of community members as research partners in rural and remote areas. Res Involv Engagem. 2020;6(1):3. Available from:<https://doi.org/10.1186/s40900-020-0179-6>

128. Minkler M. Community-based research partnerships: Challenges and opportunities. J Urban Health. 2005;82(2 Suppl 2):ii3–12. Available from:<https://doi.org/10.1093/jurban/jti034>

129. Ball J, Janyst P. Enacting research ethics in partnerships with Indigenous communities in Canada: “Do it in a good way.” J Empir Res Hum Res Ethics. 2008;3(2):33–51. Available from:<https://doi.org/10.1525/jer.2008.3.2.33>

130. Wahbe TR, Jovel EM, García DRS, Llagcha VEP, Point NR. Building international Indigenous people’s partnerships for community-driven health initiatives. EcoHealth. 2007;4(4):472–88. Available from:<https://doi.org/10.1007/s10393-007-0137-x>

131. Jull J, Giles A, Graham ID. Community-based participatory research and integrated knowledge translation: Advancing the co-creation of knowledge. Implement Sci. 2017;12(1):150. Available from:<https://doi.org/10.1186/s13012-017-0696-3>

132. de Crespigny C, Emden C, Kowanko I, Murray H. A ‘partnership model’ for ethical Indigenous research. Collegian. 2004;11(4):7–13. Available from:<https://doi.org/10.1016/S1322-7696(08)60468-0>

133. Nguyen T, Graham ID, Mrklas KJ, Bowen S, Cargo M, Estabrooks CA, et al. How does integrated knowledge translation (IKT) compare to other collaborative research approaches to generating and translating knowledge? Learning from experts in the field. Health Res Policy Syst. 2020 Mar 30;18(1):35. Available from:<https://doi.org/10.1186/s12961-020-0539-6>

134. Pelletier CA, Pousette A, Ward K, Fox G. Exploring the perspectives of community members as research partners in rural and remote areas. Res Involv Engagem. 2020;6(1):3. Available from:<https://doi.org/10.1186/s40900-020-0179-6>

135. Ramanadhan S, Ganapathy K, Nukala L, Rajagopalan S, Camillus JC. A model for sustainable, partnership-based telehealth services in rural India: An early process evaluation from Tuver village, Gujarat. Zúniga-González CA, editor. PLoS One. 2022;17(1):e0261907. Available from:<https://doi.org/10.1371/journal.pone.0261907>

136. Lim CS, Follansbee-Junger KW, Crawford MS, Janicke DM. Treatment outcome research in rural pediatric populations: The challenge of recruitment. J Pediatr Psychol. 2011;36(6):696–707. Available from:<https://doi.org/10.1093/jpepsy/jsr018>

137. MacLeod M. Building bridges with decision-makers: Rules for rural and remote health researchers. Rural Remote Health. 2006. Available from:<https://doi.org/10.22605/rrh567>

138. Oster R, Lightning P. Commentary: Developing relationships through trust in Indigenous health research. Healthc Policy. 2022;17(4):56–62. Available from:<https://doi.org/10.12927/hcpol.2022.26825>

139. Ross LF, Loup A, Nelson RM, Botkin JR, Kost R, Smith GR, et al. The challenges of collaboration for academic and community partners in a research partnership: Points to consider. J Empir Res Hum Res Ethics. 2010;5(1):19–31. Available from:<https://doi.org/10.1525/jer.2010.5.1.19>
